# Genomic surveillance reveals antibiotic resistance gene transmission via phage recombinases within sheep mastitis-associated *Streptococcus uberis*

**DOI:** 10.1186/s12917-022-03341-1

**Published:** 2022-07-07

**Authors:** Ben Vezina, Maria Nives Rosa, Antonella Canu, Sebastiana Tola

**Affiliations:** 1 Department of Infectious Diseases, Central Clinical School, Monash University, Melbourne, VIC Australia; 2 Istituto Zooprofilattico Sperimentale della Sardegna “G. Pegreffi”, Via Duca degli Abruzzi 8, 07100 Sassari, Italy

**Keywords:** *Streptococcus uberis*, Whole-genome sequencing, Horizontal gene transmission, MLST, Virulence, Antibiotic resistance, Vaccine targets, Bacteriophage, Phage, Mastitis

## Abstract

**Background:**

*Streptococcus uberis* is one of the main causative agents of ovine mastitis, however little is known about this global, environmental pathogen and its genomic mechanisms of disease. In this study, we performed genomic analysis on 46 *S. uberis* isolates collected from mastitis-infected sheep in Sardinia (Italy).

**Results:**

Genomes were assigned into lineage clusters using PopPUNK, which found 27 distinct isolate clusters, indicating considerable genetic variability consistent with environmental isolates. Geographic trends were identified including regional linkage of several isolate clusters. Multi-locus Sequence Typing (MLST) performed poorly and provided no new insights.

Genomes were then screened for antimicrobial resistance genes, which were compared to phenotypic resistance profiles. Isolates showed consistent phenotypic resistance to aminoglycosides with variable resistance to novobiocin and tetracycline. In general, identification of antimicrobial resistance genes did not correlate with phenotypic resistance profiles, indicating unknown genetic determinants. A multi-antimicrobial resistance cassette (aminoglycoside, lincosamide and streptogramin) was identified in the chromosome of three genomes, flanked by vestigial phage recombinases. This locus appears to have spread horizontally within discrete *S. uberis* populations within a 40 km radius (Sassari region).

Genomes were screened for putative virulence factors, which identified 16 genes conserved between sheep and cow isolates, with no host-specific genes shared uniformly across all host-specific isolates.

Pangenomic analysis was then performed to identify core genes which were putatively surface-exposed, for identification of potential vaccine targets. As all genomes encoded sortase, core genes were screened for the sortase cleavage motif. Of the 1445 core *S. uberis* genes, 64 were putative sortase substrates and were predominantly adhesins, permeases and peptidases, consistent with compounds found within ruminant milk such as xanthine, fibronectin and lactoferrin.

**Conclusions:**

This study demonstrated the importance of whole genome sequencing for surveillance of *S. uberis* and tracking horizontal acquisition of antimicrobial resistance genes, as well as providing insight into genetic determinants of disease, which cannot be inferred from the MLST schemes. Future mastitis surveillance should be informed by genomic analysis.

**Supplementary Information:**

The online version contains supplementary material available at 10.1186/s12917-022-03341-1.

## Background

Mastitis is one of the most common and costly diseases affecting dairy sheep. Sardinia, an island located in the middle of the Mediterranean Sea, has approximately 3.5 million milking Sarda sheep, corresponding to half of the total Italian stock. A relevant part of the regional economy relies on dairy sheep farming, mainly for pecorino cheese production. Therefore, udder health is a critical for prevention of intra-mammary infections such as mastitis, which impacts dairy yield and sheep welfare [1]. Infectious mastitis outbreaks of small ruminants can be caused by a wide variety of bacterial species, mostly *Staphylococcus* and *Streptococcus* species [2–5]. In a recent surveillance study of sheep mastitis in Sardinia, *Streptococcus uberis* was the most frequently isolated pathogen from sheep and goat milk samples [6]. *S. uberis* is considered an environmental pathogen [7, 8] which has been isolated from environmental samples, milking machines, milkers’ hands and skin of mammary teats [2, 9], making pathogen control impractical.

Current flock management procedures include hygiene, biosafety measures, proper milking, equipment maintenance, regular monitoring of animals, as well as antibiotic treatment or elimination of mastitis-positive animals [9, 10]. Vaccination is a preventative strategy which should reduce the susceptibility of dairy sheep to *S. uberis*-caused mastitis and decrease the use of prophylactic or therapeutic antibiotic treatment, as recommended by the guidelines for the prudent use of antimicrobials in veterinary medicine (2015/C 299/07). In Italy, there are currently no vaccines for immunisation of small ruminants against *S. uberis*. The Istituto Zooprofilattico della Sardegna is authorized by the Italian Health Ministry (Ministerial Decree n°287/1994) to produce an inactivated autogenous vaccine based on the *S. uberis* isolate involved in the outbreak, with the aim of limiting infection spread within the flock. However, multi-subunit epitope vaccines are preferred over inactivated whole-cell and whole antigen vaccines for eliciting a specific immune response against viral and bacterial pathogens [11]. Effective vaccination should consider the natural *S. uberis* population circulating in the geographic area, or instead target core genes shared by all isolates. Therefore, the characterization of the *S. uberis* population in each site or area is as crucial as its global epidemiology, and it is essential for the prevention and surveillance of local outbreaks. Several studies have focused their attention on single aspects, such as genetic diversity of the *S. uberis* populations, or virulence and resistance profiles [7, 8, 12–15]. Recently, whole-genome sequencing has been employed to provide in-depth analysis of *S. uberis* isolates responsible for bovine mastitis [16–19], allowing characterisation of populations, outbreak detection, putative virulence factors and critically, antimicrobial resistance genes.

Here we report an in-depth epidemiological and genomic investigation of 46 *S. uberis* isolates collected from sheep mastitis in Sardinia, Italy and determine the genetic diversity, screened antimicrobial-resistant genes, virulence factors, and cell-surface genes encoding conserved proteins to be considered as potential candidates for vaccine development. Furthermore, we compared the genetic diversity of *S. uberis* isolated from dairy cows and sheep to evaluate host-specificity.

## Results

### Strain typing and geographic distribution

**Fig. 1:**
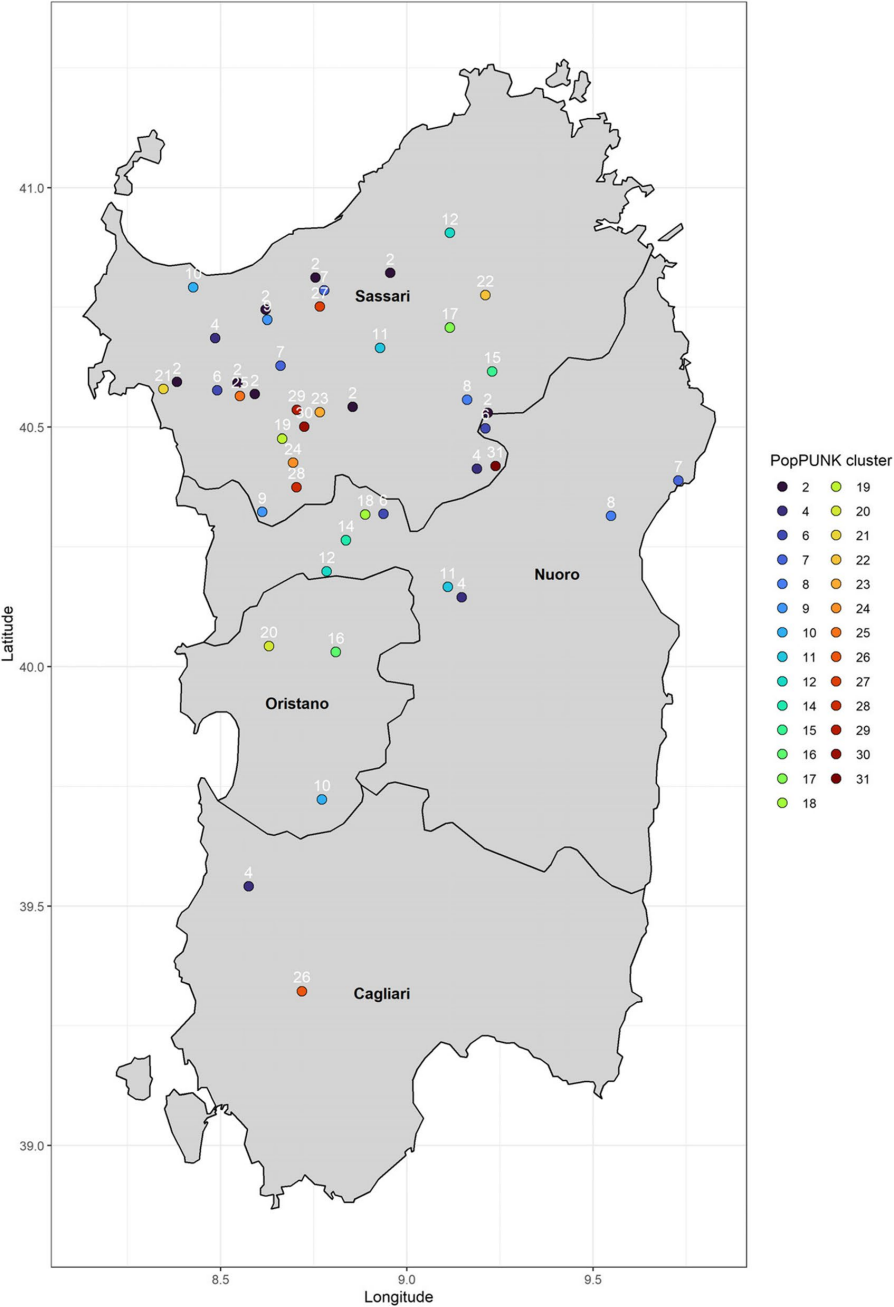
Map of Sardinia showing the collection of S. uberis isolates from sheep mastitis milk samples. Each point represents an individual isolate while point colour represents the PopPUNK cluster number as an alternative for MLST typing. PopPUNK cluster also shown in white above point. Regions are shown in bolded black text

Previously, 124 *S. uberis* strains were isolated from sheep mastitis cases across Sardinia between 2011 and 2016 [15]. For the current study, 46 of these with unique RFLP profiles were selected for whole genome sequencing because they were more representative of the municipalities with the most outbreaks. MLST analysis previously showed 84.9% of the original 124 isolates were novel STs [15]. To obtain higher resolution lineage data and leverage whole genome sequencing, PopPUNK was used to look at the relatedness of isolates, which classified all isolates in this study into 27 distinct lineages or PopPUNK clusters. Most PopPUNK clusters (18/27) were represented by a single isolate only (66%), concentrated within Sassari. However, there were some multi-isolate clusters which appeared to be geographically linked. The distribution of PopPUNK cluster 2 (eight isolates) clustered regionally within the Sassari region. PopPUNK clusters 6 and 7 (three isolates each) were also mostly geographically clustered within Sassari. A different distribution pattern was seen for PopPUNK cluster 4 (four isolates), which were distributed across four regions, spanning the entire Sardinian landmass Fig. 1.

**Fig. 2:**
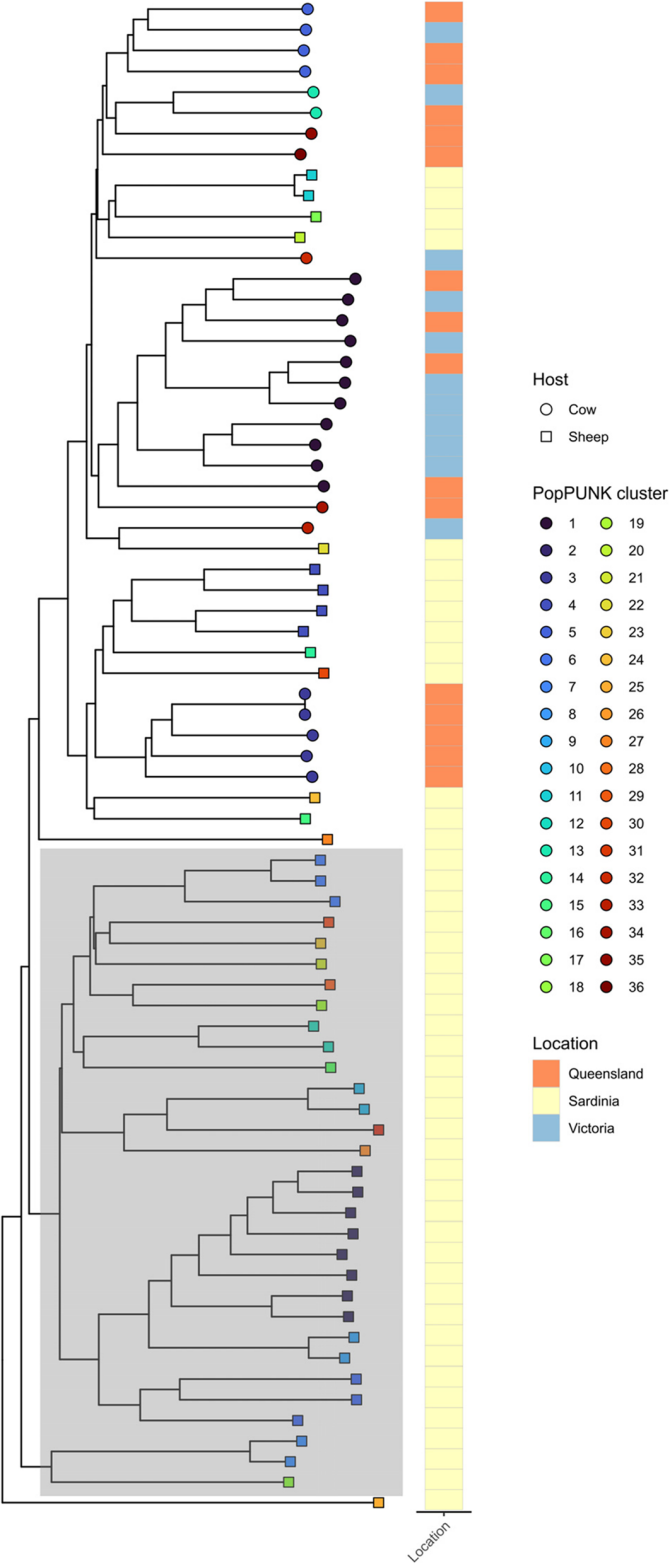
Mid-rooted neighbour-joining tree output from PopPUNK showing sheep mastitis isolates (this study) in the context of *S. uberis* from other sources. Nodes are coloured based on their assigned PopPUNK cluster, and shaped based on host. Heatmap indicates broad geographic location. Grey overlay box shows monophyletic Sardinian sheep clade

To determine if any of these isolates have any overlap with mastitis-causing *S. uberis* from other sources, these sheep isolates were compared to bovine mastitis isolates from Australia (Fig. 2).

No sheep or cow isolates shared a common PopPUNK cluster. Given the closeness of some sheep and cow isolates such as Sardinian cluster 4 (sheep) and Queensland cluster 3 (cow), regional differentiation or allopatric speciation is at least partially responsible for the genetic differences shown. Local outbreaks of same-PopPUNK clusters occur independently within these datasets, however single-isolate clusters are responsible for the overwhelming number of mastitis cases in the Sardinian dataset (Fig. 2).

### Antibiotic resistance profiles and mediation via vestigial phage recombinases

**Fig. 3:**
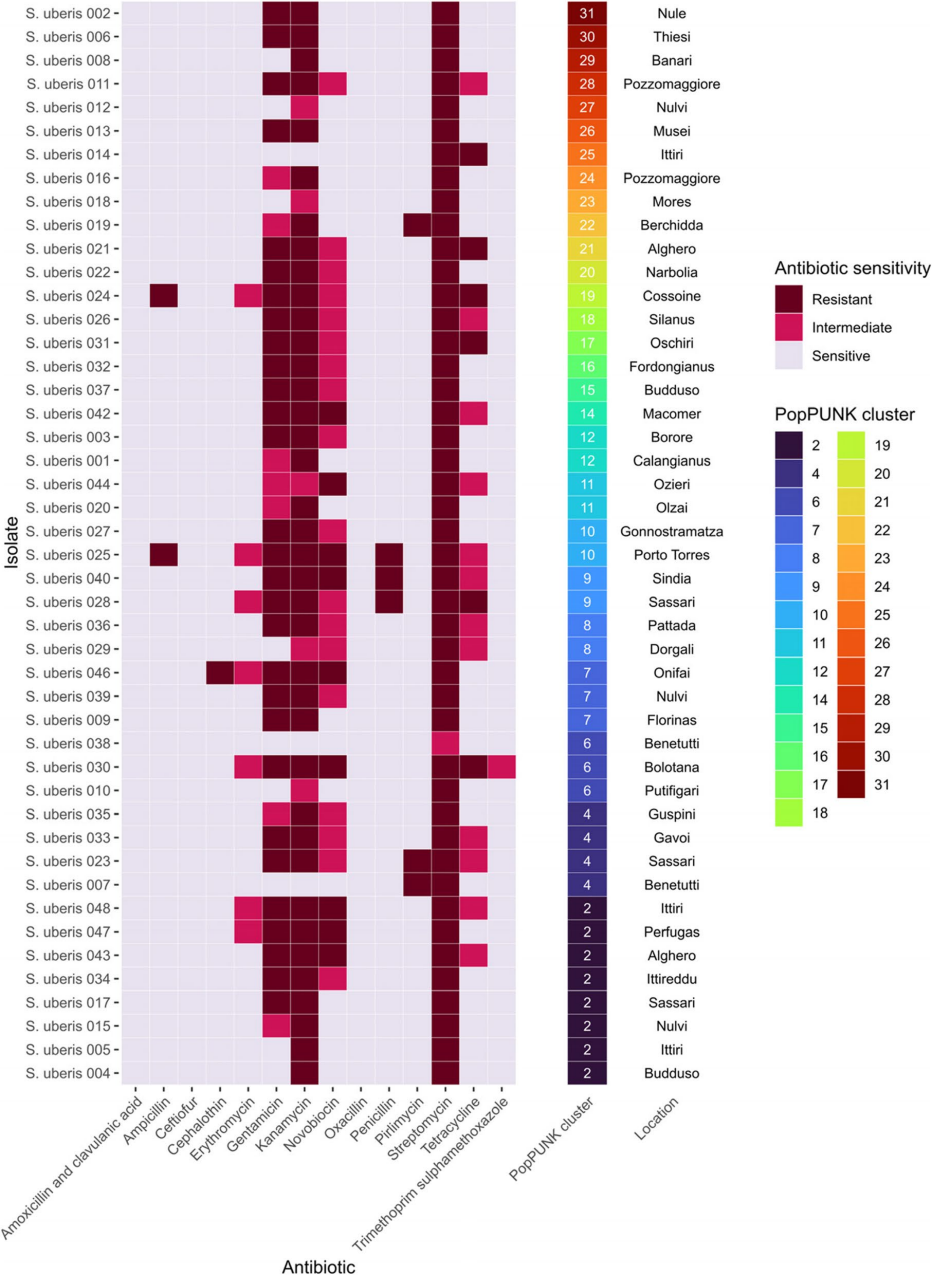
Heatmap showing antibiotic resistance profiles of all 46 *S. uberis* isolates, sorted by PopPUNK cluster. Isolates are defined as either ‘Sensitive’, ‘Intermediate’ or ‘Resistant’, reflected by increase of colour saturation. The PopPUNK clusters are annotated by the rainbow colour palette. Local location of isolate is also shown. Figure adapted from (15)

Phenotypic antibiotic resistance profiles showed high levels of resistance to aminoglycosides including gentamicin (35/46 isolates), kanamycin (43/46 isolates), streptomycin (46/46 isolates), as well as resistance to the aminocoumarin, novobiocin (27/46 isolates), as previously presented [15]. Isolates were also variably resistant to tetracycline. Phenotypic antimicrobial resistance profiles did not appear to be associated with PopPUNK clusters and therefore lineage (Fig. 3).

Antibiotic resistance gene prediction (Data S2) did not always match the phenotypic result and did not correlate strongly (Figure S1). For example, the tetracycline resistance gene *tet*(*O*) was found in three isolates (*S. uberis* 014, 021 and 027), yet tetracycline intermediate and complete resistance was found in 18 isolates. Despite *S. uberis* 027 encoding the *tet*(*O*) gene, it did not demonstrate phenotypic resistance.

The tetracycline resistance *tet*(*O*) gene was chromosomally located within the same genomic context within the three *S. uberis* isolates (014, 021 and 027), situated adjacent to the chromosomal partitioning region (*repA* and *parB*). These isolates are phylogenetically divergent (Fig. 2) and are part of different PopPUNK clusters, but have retained the *tet(O)* gene, likely from their recent common ancestor.

**Fig. 4:**
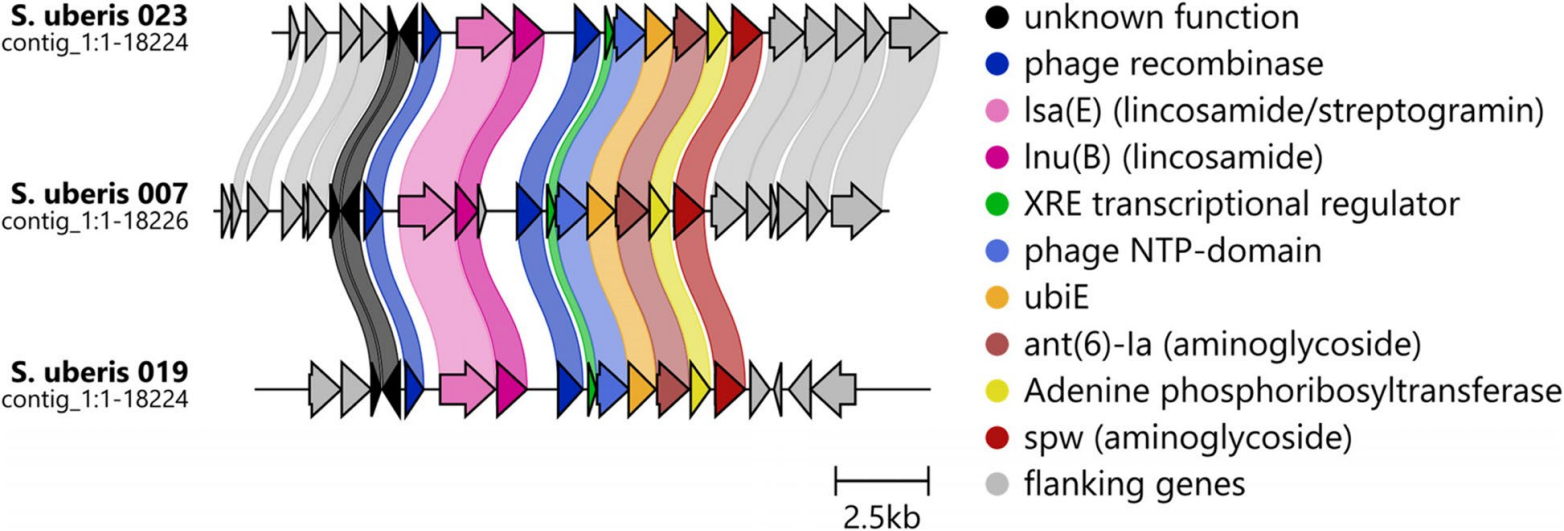
Gene cluster comparison of the phage recombinase-mediated multi-antimicrobial resistance cassette. Phage proteins are coloured in blue, with dark blue indicating the recombinase. Pink and red colours indicate antimicrobial resistance genes. Grey genes show cluster flanking genes. Black shows genes of unknown function

Four antimicrobial resistance genes *lsa(E)* (lincosamide/streptogramin resistance), *lnu(B)* (lincosamide resistance), *ant*(6)*-Ia* (aminoglycoside resistance) and *spw* (aminoglycoside resistance) were carried by *S. uberis* 007, 019 and 023. Again, this did not overtly correlate to phenotypic resistance (Figure S1). *S. uberis* 007 was one of the few isolates sensitive to kanamycin despite having two aminoglycoside resistance genes, while 41/43 of the remaining isolates lacking these genes demonstrated resistance. These four genes were found within the same locus flanked by phage recombinases (Fig. 4), shown in dark blue. The DNA recombinase has the closest hit to *Staphylococcus aureus* (accession: WP_078099357.1) while the second recombinase was closest to an *Enterococcus faecium* sequence (accession: WP_192795565.1). *S. uberis* 007 and 023 are closely related (PopPUNK cluster 4) and this multi-antimicrobial resistance cassette was found in the same genomic location. Within *S. uberis* 019 however, the cassette was found within a different chromosomal context (Fig. 4). These results indicated acquisition by the recent common ancestor of *S. uberis* 007 and 023, along with independent acquisition by *S. uberis* 019.

### Many core putative virulence genes shared between lineages

**Fig. 5:**
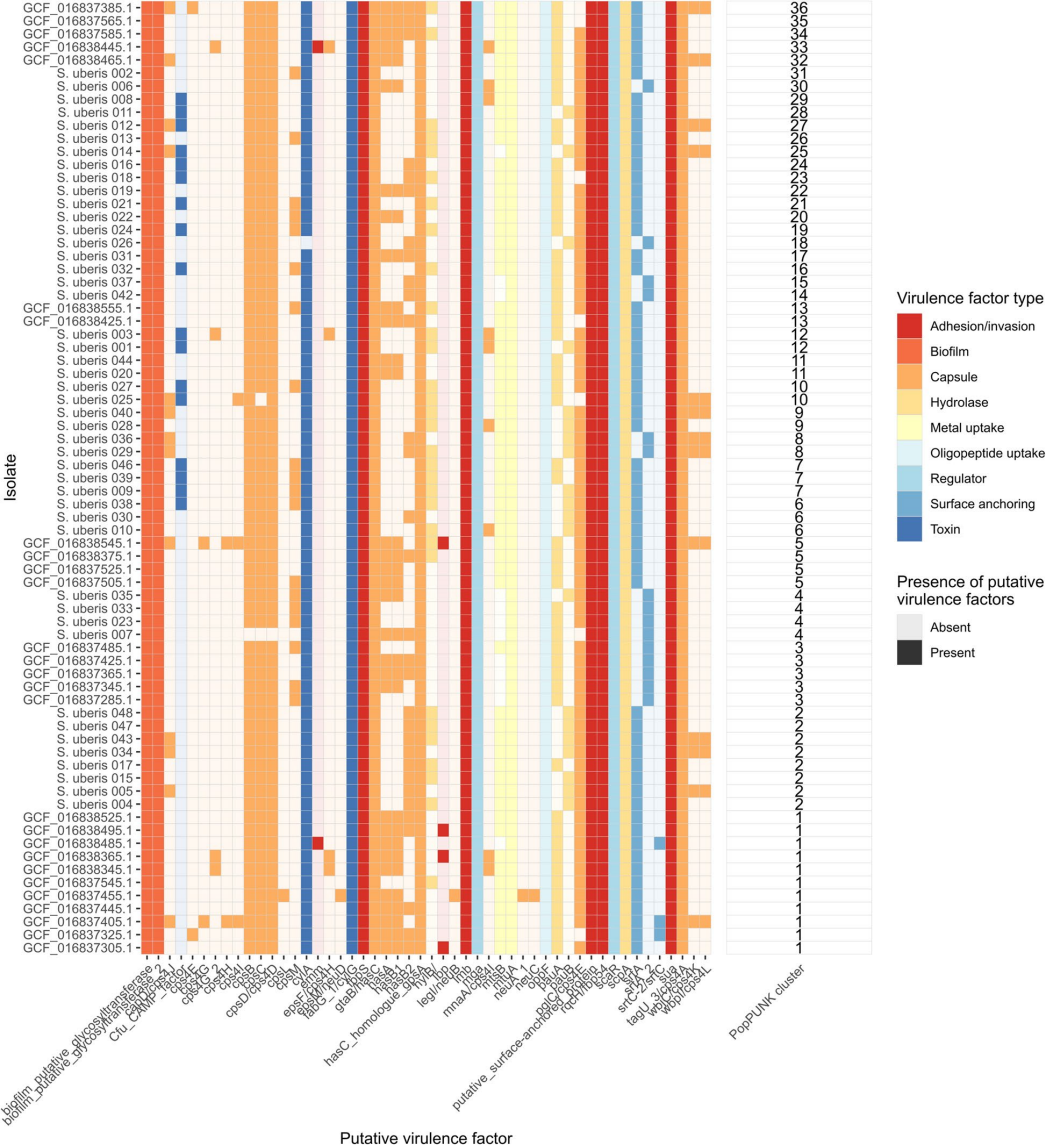
Heatmap of putative virulence factors comparing S. uberis isolated from this study to Australian isolates. Virulence factor broad ‘type’ is shown based on colour, and transparency of colour denotes presence or absence within a genome

Genomes were then screened for putative virulence factors, which found an average of 27 per genome for both sheep isolates and Australian cattle isolates (Fig. 5). *S. uberis* 003, 012, 029 and 043 had the most at 29, while *S. uberis* 007 had the least at 22. Putative virulence factors within PopPUNK clusters were not always consistent, indicating they don’t align closely with phylogeny. The exception was the two *srtA* (sortase) alleles, and either the first or second allele was present in any given cluster. Isolates from sheep and cows shared 16 putative virulence factors as core genes including at least one *srtA* allelle, *fabG 1*/*cylG*, *fbpS*, *gtaB*/*hasC, hasC* homologue *gpsA*, *lmb*, *mga*, *mtuA*, *oppF*, a putative surface-anchored protein, *rqcH*/*fbp54*, *scaR*, *scpA*, *sua* and *tagU3*/*cps4A.*

In terms of host-specific genes, 17/46 sheep isolates contained *cfu*, a CAMP factor, while none of the cow isolates did. 4/27 cow isolates exclusively had the lactoferrin binding protein *lbp*, as well as 2/27 containing the *emm* adhesion gene, absent in sheep isolates.

### Surface-exposed SrtA targets for vaccine design

Given the variety of singleton strains causing sheep mastitis, vaccine approaches are only likely to be successful if the variation in *S. uberis* can be accounted for. Also, designing vaccine targets which are surface-exposed also provides an advantage to successful vaccination. To address this, we performed pangenome analysis on sheep isolates, and analysed the core genome for SrtA cleavage motifs. As SrtA is one gene responsible for anchoring proteins to the cell surface and has been shown to be essential for bovine mastitis virulence [20]. The core genome was comprised of 1445 genes (including *srtA*), out of a total 5413 pan genes.

Of these, 296 protein-coding sequences were identified as predicted SrtA substrates. Excluding the broader LPXXXD motif, 64 matches were found to the remaining motifs (Data S4). Notable putative core sortase substrates included a xanthine/uric acid permease gene (WP_012658369.1), a PII-type proteinase (WP_042748147.1), serum opacification factor/fibronectin and fibrinogen binding protein (WP_195847669.1), a lactoferrin binding protein (AAQ83577.1), adhesins (closest hits WP_222357132.1, WP_039695954.1 and WP_060458349.1), peptidases (WP_203261209.1, KKF55867.1 and WP_043052350.1) and oxidoreductases (FAD/NAD(P) binding and NADP-dependent). These genes represent protein targets of interest for vaccine development, given their likely-surface exposure and being part of all *S. uberis* genomes isolated from sheep in this study.

## Discussion

The diversity of *S. uberis* isolates causing mastitis in Sardinian sheep surveyed in this study demonstrated that most isolates were novel sequence types (Data S1). This was corroborated by single-isolate PopPUNK clusters being responsible for the overwhelming number of mastitis cases in sheep (66%) (Fig. 1). This data is consistent with dairy animals being opportunistically colonised by environmental [7, 8] or gastrointestinal carriage of *S. uberis* [21].

Cow and sheep isolates were mostly not separated phylogenetically by any clear distance (Fig. 2). There was one large monophyletic clade which consisted entirely of 31 sheep isolates, but the specific determinants of this is unclear from this limited dataset. The putative virulence screen (Fig. 5) indicated more shared core virulence genes than host-specific virulence genes (which were found exclusively within hosts but not wholly across all isolates from these hosts). The core genome of sheep isolates was also similar in size to bovine-mastitis associated *S. uberis* [16]. These findings are consistent with colonisation by random environmental isolates as opposed to specific-host adaptation. Larger genomic surveillance efforts should be undertaken to examine this, along with examination of potential environmental reservoirs. Multiple samplings from same flocks should also be sequenced to examine outbreak cow-to-cow spread within flocks.

Unlike the single-isolate clusters, multi-isolate PopPUNK clusters 2, 6 and 7 were geographically linked within the same regions, mostly within Sassari (Fig. 1). This was not unexpected, given that 32/46 isolates were sampled from Sassari. Larger sampling efforts across all provinces would be advantageous for future regional analysis. As these isolates are not closely-related enough to be considered direct outbreak transmission, it indicates common *S. uberis* lineages are either colonising the environment within at least a 40 km radius or have spread fairly recently via animal exchange to cause mastitis across eight Sassari farms, which would possibly explain the divergence seen. Similar observations were made for PopPUNK clusters 6 and 7, which were also distributed across the same region, indicating potential competition for sheep mammary tissue. Multiple samplings from same-farms should be taken to capture if any, inter-flock competition between isolates. Finally, PopPUNK cluster 4’s distribution across the entire Sardinian landmass indicates the wide distribution of similar *S. uberis* in the environment. It is likely that high-density sampling in other regions will find similar patterns for other *S. uberis* lineages.

Antimicrobial resistance genes were rare across the 46 sequenced sheep isolates (Fig. 3). The low correlative scores between antimicrobial resistance genes and phenotypic resistance profiles (Figure S1), except in the case of *lsa(E)* and *lnu(B)*, and phenotypic pirlimycin resistance indicate the incompleteness of current antimicrobial resistance database and knowledge within *S. uberis*. This can be partly explained by false negatives, where an antimicrobial resistance gene is present but the strain is phenotypically sensitive, likely caused by a lack of gene expression, rather than resistance capability [22]. This was likely the case for *tet(O)* presence in *S. uberis* 027 despite lack of tetracycline resistance. As resistance in *S. uberis* is poorly understood and absent from databases, this underscores the importance of using whole genome sequencing in concert with phenotyping to understand genetic mechanisms of resistance.

The most reasonable explanation for the retention of the *tet(O)* gene in three phylogenetically distinct *S. uberis* isolates (three different PopPUNK clusters), is vertical inheritance from their recent common ancestor combined with continued use of tetracycline as a measure of controlling mastitis in Sardinia, allowing the gene to be retained by individuals. The *tet(O)* gene was found adjacent to the chromosomal partitioning region, *repA* and *parB*, a critical region required for effective cellular replication. This region may provide insulation from deletion events and could explain the retention of *tet(O)* across phylogenetically distinct isolates.

The phage recombinase-flanked multi-antimicrobial resistance (aminoglycoside, lincosamide and streptogramin) cassette found in the chromosomes of *S. uberis* 007, 019 and 023 (Fig. 4) demonstrated the importance of whole genome sequencing for surveillance of horizontal gene transfer through mobile genetic elements. Phages have been shown to shuttle antimicrobial resistance genes [23], however these phage recombinases are vestigial remnants as no complete phage is locally intact. This finding shows these antimicrobial resistance genes have been co-opted by these mobile genetic elements and can spread independently within discrete *S. uberis* populations. Given that *S. uberis* 007, 023 and 019 are all geographically isolated within a 40 km radius of each other within the Sassari region, a local environmental reservoir likely exists. The structure of the cassette indicates the aminoglycoside resistance portion is regulated separately from the lincosamide and streptogramin portion (due to gene spacing and upstream XRE transcriptional regulator). This is one explanation for the irregular kanamycin sensitivity of *S. uberis* 007 and intermediate sensitivity of *S. uberis* 019 to gentamicin, despite presence of two aminoglycoside genes. Alternatively, these genes encode resistance to other aminoglycosides not tested in this study.

Screening of putative virulence factors indicated a considerable number of conserved genes (Fig. 5). *hasC* has been noted as a core gene in two previous genomic studies on *S. uberis* in bovine mastitis [16, 17], which is a known virulence factor involved in capsule production and resistance to phagocytosis [24]. Also confirmed from a previous analysis [16] was the presence of at least one of two sortase (*srtA*) alleles, essential for mastitis [20]. The large number of core, putative SrtA substrates identified (Data S3) appears to corroborate this finding. The types of genes identified (adhesion and binding genes, permeases and peptidases) are consistent with compounds found within ruminant milk such as xanthine [25], fibronectin [26] and lactoferrin [27] where these isolates were cultured from. The PII-type proteinase found allows breakdown of beta-casein, a major component of milk [28].

The xanthine/uric acid permease gene (WP_012658369.1) was 99% similar to a previously identified xanthine permease gene from cow isolate[16], predicted to be involved in colonisation of the mammary gland. Lactoferrin is a soluble glycoprotein and is an innate immunity factor found in mammary glands [27], functioning as a bactericidal agent. The putative virulence factor lactoferrin binding protein (CAR40577.1) which was found exclusively in 4/27 cow isolates also contained a SrtA LPXTG cleavage motif and may be related to overcoming the innate immune response and allowing colonisation establishment by *S. uberis*. Larger sampling efforts and genomic analysis including Genome Wide Association Studies and mutagenesis libraries will be required to fully understand the extent of the host-specific virulence genes, if any.

We find the abundance of new publications identifying ‘novel’ MLSTs causing mastitis, which this study also found, troubling. *S. uberis* is an environmental pathogen which has a diverse number of ‘novel’ lineages capable of causing disease, rather than acquisition of consistent, problematic clones. MLST typing in *S. uberis* does not provide new insights into pathogenesis or epidemiology. Moreover, some isolates are lacking ‘core’ genes used as typing genes, as found in this study and in previous genomic analyses [13]. Phylogenetic trees built comparing the conserved core genome between isolates (using thousands of genes rather than the 7 genes used for MLST) do not cluster STs together which would be expected for a robust and reliable MLST typing scheme consistent with the population structure, as noted in a previous study [16].

Instead, we propose future study of this environmental pathogen should involve genomic surveillance and analysis of genetic relatedness directly in context of geographic distribution, along with analysis of genes involved in antimicrobial resistance and pathogenesis. Future study should also include identification of natural reservoirs of *S. uberis*, to improve flock management practices and administration of appropriate antibiotics. This analysis carried out on a larger scale will lead to identification of suitable vaccine targets, which is the cheapest and most effective way to control *S. uberis*-associated sheep (and bovine) mastitis.

## Conclusions

In this study, we used whole genome sequencing and analysis to analyse sheep mastitis-associated *S. uberis*. In general, antimicrobial resistance genes were uncommon, though a vestigial bacteriophage recombinase-flanked resistance cassette was identified in three distinct but geographically co-located isolates, indicating horizontal mobilisation. We identified 16 putative core virulence factors were shared between cow and sheep isolates associated with mastitis which may contribute to these environmental isolates ability to cause disease, along with surface-exposed core genes that are consistent with compounds found abundantly within milk. We also identified key gaps in current knowledge such as unknown genetic determinants of antimicrobial resistance. Finally, we advocate for a genomics-based approach for future analysis of mastitis-associated *S. uberis* instead of reporting MLST, which does not provide novel insight or have robustness in the context of population structure.

## Methods

### *S. uberis* collection

A total of 46 *S. uberis* isolates were analysed for this study. Isolates (one per farm) were randomly selected from a bank of *Streptococcus* species used for the preparation of inactivated autogenous vaccine, due to the presence of intramammary infection in the flock. Information about the type of mastitis was not provided by the farm veterinarian. The geographic distribution of these isolates in the Sardinian land is reported in Supplementary Figure S1. Bacterial identification at the species level was determined by PCT-RFLP and MALDI-TOF MS, as described in a previous study [2].

### DNA extraction, library preparation, and sequencing

*S. uberis* isolates were cultivated in blood agar for 24 h at 37°. Freshly overnight colonies were picked up, suspended in 5 mL of Brain Heart Infusion broth (BHI, Oxoid LTD, Basingstoke, UK), and grown at 37 °C for 18 h with shaking. Cultures were harvested by centrifugation at 5,000 x g for 10 min. DNA was extracted from the pellet using DNeasy Blood and Tissue kit (Qiagen, Valencia, CA, USA) following the protocol designed for Gram-positive bacteria with the only modification of the use of 250 µL distilled water for the elution step. Eluates were concentrated until one-tenth of the original volume using Amicon Ultra 0.5 mL centrifugal filters at 6,000 x g for 30 min. DNA was quantified using a Qubit fluorometer (Invitrogen Corp., Carlsbad, CA, USA). Sequencing libraries were made using Ion Xpress Plus Library kit (Thermo Fisher Scientific, Frederick, MD, USA), according to the manufacturer’s instructions. Libraries were sequenced with an IonTorrent Personal Genome Machine (PGM) (Life Technologies, Carlsbad, CA) at the Istituto Zooprofilattico della Sardegna.

### Antimicrobial susceptibility testing

Antimicrobial susceptibility testing was performed through the disc diffusion method on Mueller Hinton agar supplemented with 5% of defibrinated sheep blood using an inoculum corresponding to the 0.5 McFarland standard. The plates were incubated at 37 °C for 24 h in an atmosphere of 5% CO2 before measuring the zone of inhibition. The following antibiotic discs (Oxoid, Basingstoke, UK) were used: streptomycin (S, 10 µg), kanamycin (K, 30 µg), gentamicin (CN, 10 µg), ampicillin (AMP, 10 ug) penicillin (P, 10 IU), amoxicillin-clavulanic acid (AMC, 30 µg), oxacillin (OX, 5 µg), tetracycline (TE, 30 µg), erythromycin (E, 15 µg), trimethoprim-sulphamethoxazole (SXT, 25 µg), cephalothin (KF, 30 µg), novobiocin (NV, 30 ug), ceftiofur (EFT, 30 ug), and pirlimycin (PIR, 2 ug). S. pneumoniae ATCC 49,619 were used as the quality control strain. Isolates were classified as susceptible, intermediate or resistant based on inhibition zone diameters, according to guidelines of the Clinical and Laboratory Standards Institute [29]. For NV, EFT and PIR, the susceptibility breakpoints were based on *S. uberis* collected from bovine mastitis. For the remaining antimicrobials, the susceptibility categorization was based on human Streptococcus-derived breakpoints. Multidrug resistance (MDR) was defined as resistance to ≥ 3 antimicrobial classes.

### Assembly

Spades [30] version 3.15.3 was used to assemble the IonTorrent reads with the following options: ‘--iontorrent --isolate’. K-mers were iterated through starting with -k 27, then 53,71,87,99,111,119,127 with the ‘--restart-from k[53…127]’. After these 8 assemblies were complete, the .gfa files were evaluated using getUnicyclerGraphStats.py from Unicycler version 0.4.7 [31] and the assemblies with the fewest GFA dead ends, then fewest contigs were chosen as the best assembly.

### Annotation

Bakta version 1.1.1 [32] was used to annotate genomes using Bakta database (date accessed: 1/09/2021).

### Pangenomic analysis

The annotated genomes analysed using Panaroo version 1.2.8 [33] using the following options: ‘--clean-mode sensitive -a core --aligner mafft --no_clean_edges --core_threshold 0.98 --merge_paralogs --remove-invalid-genes’.

These were run separately on Panaroo. The Panaroo output graphs for all species were then merged using the panaroo-merge command with the following options: ‘--merge_paralogs’ to obtain the final pangenome.

### Identification of putative SrtA substrates

The pangenome was translated using transeq from EMBOSS version 6.6.0 [34] with the following options: ‘-Tables 11 -frame 1’, then the core genome was extracted. This fasta file was then screened for several SrtA motifs including LPXTG [35], LPXXXD [36], LPXTA [37], QVPTGV and LPSTGE [38] using GNU grep version 2.20 with the following command: “egrep -B 1 ‘LP.TG|LP…D|LP.TA|QVPTGV|LPSTGE’”.

### Antibiotic resistance screening

AMRFinderPlus version 3.10.23 [39] was used to screen assemblies for antibiotic resistance genes using protein and gfa inputs with identity set to 80%: ‘-i 0.8’.

### Virulence screening

Abricate version 1.0.1 [40] was used to identify putative virulence factors using a custom *S. uberis* putative virulence factor database [16]. Cut-off values of 0.95 for nucleotide identity and 0.9 for coverage were used.

### Multi-locus sequence typing

mlst version 2.19.0 [41] was used to classify strains within the *S. uberis* Multi-Locus Sequence Typing (MLST) scheme [42], using the ‘--scheme suberis’ option.

### Isolate clustering

PopPUNK version 2.4.0 [43] was used to identify clusters within isolates. The ‘create-db’ function was used with the following options: ‘--sketch-size 1000000 --min-k 15 --max-k 29 --qc-filter prune’. Then the ‘fit-model’ function was used with the following options: ‘bgmm --k 3 --graph-weights’. The rank 1 clustering was chosen, then ‘poppunk_visualise’ function was used, with the ‘--distances’ and ‘--previous-clustering’ utilising the refined model fit, to output a neighbour-joining core tree.

### Data visualisation

Data were visualised using R version 4.0.3 [44] and RStudio version 1.3.1093 [45] with the following software packages: RColorBrewer version 1.1-2 [46], viridis version 0.6.2 [47] tidyverse version 1.3.1 [48], ggplot2 version 3.3.3 [49], reshape2 version 1.1.4 [50] and aplot version 0.0.6 [51]. ape version 5.5 [52] ggtree version 2.4.1 [53] was used for phylogenetic tree visualisation only. Map was constructed using ggmap version 3.0.0 [54], maps version 3.4.0 [55], mapdata version 2.3.0 [56].

Clinker version 0.0.23 [57] was used to generate genome cluster comparison figures.

## Supplementary Information

**Additional file 1: Figure S1.** Correlation matrix comparing presence of antibiotic resistance gene (y axis) to phenotypic resistance profiles (x axis). Presence of resistance gene = 1, absence = 0. Phenotypic growth profiles were also scaled by converting ‘Resistant’ to 1, ‘Intermediate’ to 0.5, and ‘Sensitive’ to 0.

**Additional file 2: Data S1.** Strain data and associated metadata of all isolates used in this study.

**Additional file 3: Data S2.** Antimicrobial resistance information for each strain. Antimicrobial resistance gene presence is shown along with phenotypic resistance profiles.

**Additional file 4: Data S3.** Putative virulence factor screen for each strain.

**Additional file 5: Data S4.** Fasta file containing all putative SrtA substrates which are also core genes.

## Data Availability

All data generated or analysed during the study are included within the article and supplemental material. Reads and assemblies generated in this study have been uploaded to Genbank under BioProject accession: PRJNA826207. Each BioSample accession can be found in Data S1.

## Abbreviations

BHI: Brain Heart Infusion
MDR: Multi-Drug Resistance
MLST: Multi-Locus Sequence Typing
