# Supplementary figures and images for: Genomic surveillance reveals antibiotic resistance gene transmission via phage recombinases within sheep mastitis-associated Streptococcus uberis
Source: BMC Vet Res. 2022 Jul 7;18:264. doi: 10.1186/s12917-022-03341-1 (PMC9261030; doi:10.1186/s12917-022-03341-1)

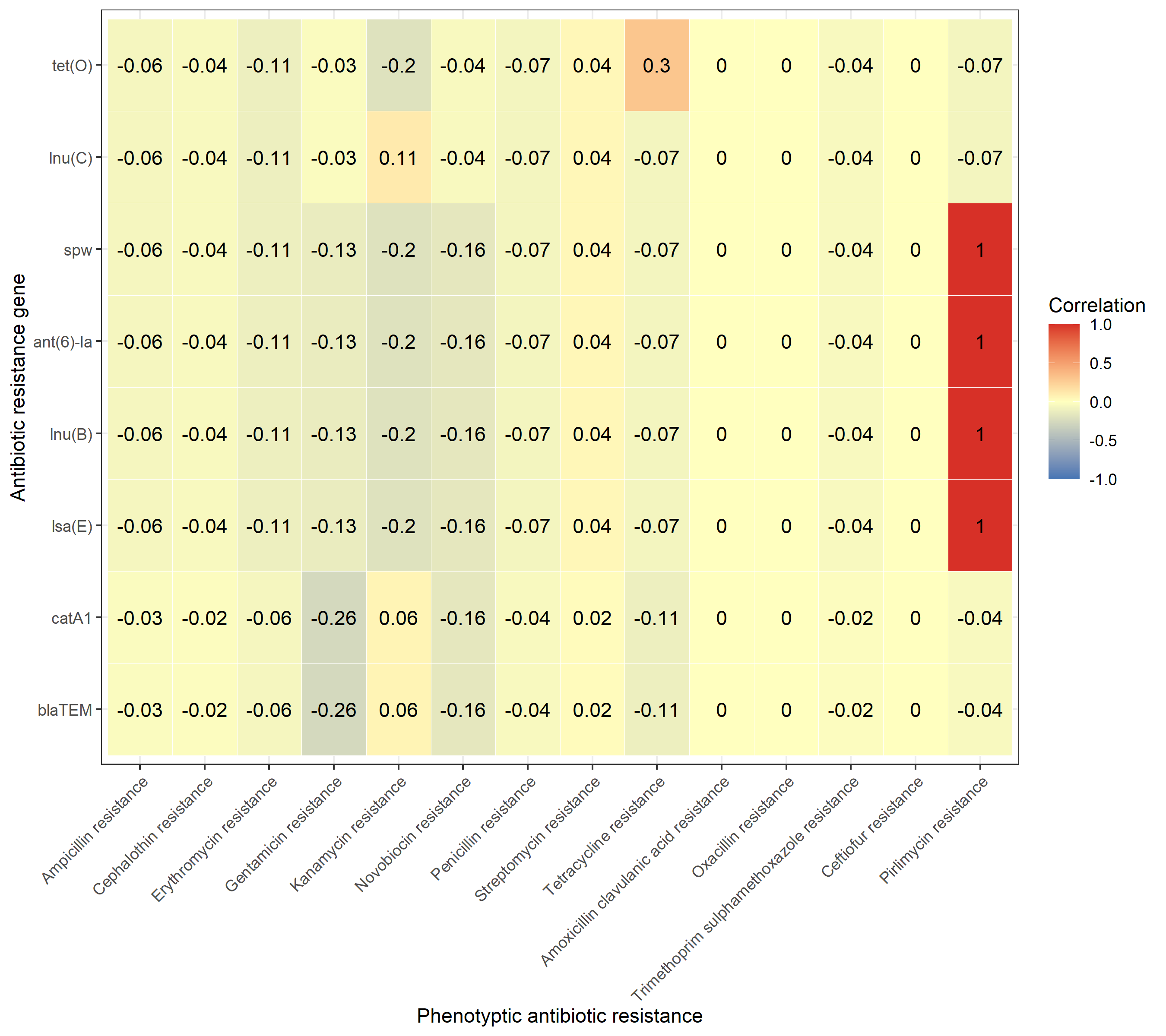

Supplement: Supplementary file 1 — Additional file 1: Figure S1. Correlation matrix comparing presence of antibiotic resistance gene (y axis) to phenotypic resistance profiles (x axis). Presence of resistance gene = 1, absence = 0. Phenotypic growth profiles were also scaled by converting ‘Resistant’ to 1, ‘Intermediate’ to 0.5, and ‘Sensitive’ to 0. [file 12917_2022_3341_MOESM1_ESM.png]
