# Supplementary material for: Genomic surveillance reveals antibiotic resistance gene transmission via phage recombinases within sheep mastitis-associated Streptococcus uberis
Source: BMC Vet Res. 2022 Jul 7;18:264. doi: 10.1186/s12917-022-03341-1 (PMC9261030; doi:10.1186/s12917-022-03341-1)
Supplement: Supplementary file 5 — Additional file 5: Data S4. Fasta file containing all putative SrtA substrates which are also core genes. [file 12917_2022_3341_MOESM5_ESM.pdf]

>ileS
MKLKETLNLGKTAFPMRAGLPNKEPLWQAAWDEAQIYQKRQKLNEGKPSFHLHDGPPYANGNIHVGHALNKIS
KDIIVRAKSMSGYNAPYVPGWDTHGLPIEQVLAKKGIKRKEMDLAEYLEMCRDYALSQVDKQRQDFKRLGVSA
DWDNPYVTLDPAYEADQVRVFGAMADKGYIYRGAKPVYWSWSSESALAEAEIEYHDIDSTSLYYANKVKDGKG
ILDTDSYIVVWTTTPFTVTASRGLTVGPDMDYVLVQPASTSKKYLVAEGLLDSLAPKFAWTDFEILSHHKGSE
LEYIVTEHPWDSDVDELVILGDHVTLDSGTGIVHTAPGFGEDDYNVGTKYKLEVAVTVDERGMMMENAGPDFQ
GQFYDKVLPTVIEKLGDLLLAKEVINHSYPFDWRTKKPIIWRAVPQWFASVSDFRQEILDEIEKTTFHPSWGK
TRLYNMIRDRGDWVISRQRAWGVPLPIFYAEDGTAIMTKEVTDHVANLFEKEGSIIWWKKEAKELLPEGFSHP
GSPNGEFTKETDIMDVWFDSGSSWNGVMNAREQLSYPADLYLEGSDQYRGWFNSSLITSVAVNGHAPYKAVLS
QGFVLDGKGEKMSKSKGNIISPNDVAKQYGAEILRLWVTSVDTDNDVRVSMDILGQVSETYRKIRNTLRFLIA
NTSDFNPNTNRVAYEELAPVDKYLTIKFNKLVEVFNNSYENYDFMAIYKAVVNFVTIDLSAFYLDFAKDVVYI
EAANSLARRRMQTVFYDILVKITKLLTPILPHTAEEIWSYLEFEEEEFVQLTEMPVAEIFEGEEDILSAWDAF
MTLRTQAQKALEEARNEKVIGKSLEAHLTIYASDEVKTLLTALDSDIALLMIVSQLTIADFADAPTNAISFEN
VAFTVDHAVGEVCERSRRVDPTTRMRSYGAFVCDASAKIIEENFPEAVAQGFESN*
>pbuX
MQTETQHSHSQSAILGLQHLLSMYAGSILVPIMIAGALGYSAKELTYLISTDIFMCGLATFLQLQFNKYFGVG
LPVVLGCAFQSVAPLSIIGAKQGSGAMFGALIASGLFVILIAGVFSKIARFFPAIVTGSVITTIGLSLIPVAM
GNMGNNTPKPTGQSLILAFLTIFIILAIQKFATGFIKSIAILIGLIAGTLVAALMGLVDTSAVSSAPWVHVPT
PFYFGAPKFEITSIVMMCIIAIVSMVESTGVYLALSDITDETLDSNRLRNGYRAEGLAVLLGGIFNTFPYTGF
SQNVGLVRLSGIKTRRPIYYTAAFLVVIGLLPKFGALAQMIPSPVLGGAMLVLFGMVALQGMQMLNRVDFQGN
EHNFIIAAVSISAGVGFNGTNLFASLPSTANMFLTNGIVIATVTAVLLNLIFNGNTKEN*
>ftsY
MGLFDKLFGKKTKEQQDEKDQVENELGVDSSFPEESQAEEQNIFEKPANSQHIEEELSLASDPASIEEVIIQQ
QELTQSENLETVIEGDHLDNKDIQGENSDLERQEQVSDHADHQLDNLVENAEENQEQELSTEKTYSIVDEYYQ
RKAAFEEGLQNGDFEPLSQNDITPSPKPQLEESDQEKYQRSLKKTRNGFAARLNAFFANFRSVDEDFFEELEE
MLILSDVGVSVATELTEELRQEAKLENAKKPDDLKRVIIEKLVDIYEKDGTFNEAINLQEGLTVMLFVGVNGV
GKTTSIGKLAYRYKAEGKKVMLVAADTFRAGAVAQLAEWGRRVDVPVVMGAEKADPASVVFDGVEKAVAQGVD
ILLIDTAGRLQNKENLMAELEKMGRIIKRVIPDAPHETLLALDASTGQNALSQAKEFSKILPLTGLILTKIDG
TAKGGVVLAIRQELEIPVKFIGFGEKIDDIGEFDSEDFMKGLLADII*
>group_415
MRKFLMSCFAALLLLFAGVSQADADQYLRVGMEAAYAPFNWTQDDNSNGAVPIEGTNQYANGYDVQVAKKVAK
SLNKKLLVVKTSWTGLIPALTSGKIDMIAAGMSPTEERKKEIAFSDSYYTSEPVIVVKADSKYAKAKSLDDFA
GAKITAQQGVWHVNLIPQINGVKAQTPMGDFSQMRQALSSGVIDGYISERPEAMTAENANSAFKMVVLKKAFT
VNESDAAIAVGMRKDDPRIVQVNTVLADLSANDRLDLMDKMVTLQPKEKKAENGAQPSFLDQMWSIVTKNWKQ
FLRGTGLTLLISTIGTIVGLIIGLLIGIYRTAPKSKHKVLAFFQKLFGWFLNVYIEVFRGTPMIVQSMVIYYG
TAQAFGISIDRTLAAIFIVSINTGAYMTEIVRGGIFAVDKGQFEAATALGFTHGQTMRKIVLPQVVRNILPAT
GNEFVINIKDTSVLNVISVVELYFSGNTVATQNYQYFQTFSVIAVIYFILTFTVTRILRYVERRIDDDNYTTT
VNELP*
>group_704
MKVEIIERNNKKVLKKTKKDGSISYTIKGAYLGIDKKTGKQVTTTISAPTLKALDRKYQDVRREFEENDSTRK
ETAEIGTIQDLAEIWFDSYKTWVSSDNTRNKVRGYLDTYIIPRFGDYKIDAIESTEVQQWVDSLAEQARSEVA
KGKGKAEKGKASDYGAVVHKLIDILDFGMVHFNLKFNSAKQVRIPPKPRAKNKRVRVLHEKDLAKWLNYLDTL
PNTRANRRFKIICNTLLASALRINELLALEIDDLHIDTNEIEVNKTLMWRSANKKLGTKGKVVCKNSAKTESG
NRRITVPESIMQDLLNIHNEMNEYFKKHNLPESKLIFPTIYGNYMTDRNERATLIKRLKSLGLPDTGFHLFRH
THASLMLNSGADWKELQERMGHKSISTTMDIYAELDPNRKNEAVDILLEKLSDIKNSN*
>group_2949
VISSTPTNGLSVDENGNLVGTPTVDDWGTDEETREVKVPVKVTNTLPDGTEEVVEVEVPVTVNRDTDGDGIID
SEDPDDDNDGVEDGSDDTPKAFTGFTATAKPVTVPEGQAVPADTKVNTLPLTGDSSEAFFTSAALAIISSVGL
LGIAKGKKNGSD*
>group_241
MLEIKIAVNDPQIMAQDGSSILRSLQNKSLPLVDLIVRESIQNSLDATLEESDTTKVDIKVDKFDTLTCSNHF
QGIETELKEKYLNNNYFLSIRDSNTSGLSGKVIGESIAELNDSNFFKLVFALGKNQEKAGSGGSWGLGKTSYF
RIGNGIVIYYTRVKSETGYEERLIASLIEDNKEEDRLLKNSDRGIAWWGKINNEDVILPITNSDEISEILSIF
KIRQYVGTETGTTVIIPYLSNEFIHRESTKNQIEKSCFWLNDLSEEIKIAVQRWYFPRLNNDTYRLHFNQSKL
ICSINDSLEINYFPEVTFKLLQNIYNSALLGNTIDENTINVYPIRLPRKGAVDVNKPIGHIAFMEVSKEDAKM
LPPDNLQSPLAYLGNYDEKQNTEYKSKFLCYCRKPGMIIEYDFTDNWLPNGNIQKENHLLFSVFVPNSDLELS
QTTYKTYPNVENYLRSIENADHAKWEDEHGYTFVKRTKSYIIDTVKKHFDNDENSTVGAATNRLSKILGNALL
PPTGFGKRSSRVVTGNSNINRNSSSKASISVEDVSFVDFKTVKVLVKGRIIKNRKTKIKILVETQEGKIDKDR
WEGDLNTIDFPFIITSAKFINDNFKLQEDYIVEGNCILINELPTDKNIEVEILINKSTNQYSPVISITS*
>group_4349

MTKDELIARLRSLGEQLNRDVSLTGTKEELALRVAELKEELDDTDETAGQDTPLSRENVLTGHENEVGSAQPD
TVILDTSELVTVVALVKLHTDALHATRDEPVAFVLPGTAFRVSAGVAAEMTERGWPECNNGSAVADSITCSML
PLPAPMKRYAGTWERQPPLHPVSSQVR*
>group_4355
LQAAKQELTNLIASAKTLSASGKYDDATTTALAAATQKAQTALDQTNASVDSLTGANRDLQTAINQLAAKLPA
DKKTSLLNQLQSVKAALGTDLGNQTDPSTGKTFTAALDDLVAQAQAGTQTDDQLQATLAKVLDAVLAKLAEGI
KAATPAEVGNAKDAATGKTWYADIADTLTSGQASADASDKLAHLQALQSLKTKVAAAVEAAKTVGKDDTTGTS
DKGGGQGTPAPAPGDTGKDKGDEGSQPSSGGNIPTNPATTTSTSTDDTTDRNGQLTSGKGALPKTGETTERPA
FGFLGVIVVSLMGVLGLKRKQREE*
>group_4438
VPVPVVTTRTEVIAHTTKYIADDTLAYGQRQTVVAGVDGEKTYTTTDGVEDAGVVTKAMVAEEIRVGTKPTVE
TVTIAKPADVEEADATLAKGTRVLKTAGSTGSTTTTTTYSMDATTGVVTANTPTVETVAASPDVYRVGTKEDV
IEIVEEIVEKTKSFGKVVINNPDLLKGISRQVRLGKNGIIEVTYKIVKVNGLEISREIISEKEIEKPVDEIVE
IGTKEPVENQNQKVIKANESKMNSTDVKKVENKGELPKTGEIKESVFGMFSVIMTGLFALVTFLGKRERK*
>group_1680
MKNITKIITLTSVVALTSLAPLDIFADQVDSTTPSTGVVVPGDIAPSTPVPGDTTTPSIDPSTPVPGDTTTPS
IDPSTPVPGDATTPSIDPSTPGSGDATTPSIDPSTPGSGDTTAPSSDPNTPKPEDTTSPSTSEPGDTSVPVTP
GIIDKVDPNTGSVTIKPIPVNPGTSVIGTQDGNLIVLDNEGKQSIVSAESLGGRVNNNGTVTIKDAEGKEKTL
PHTGQETTFIVQLIGFFSILISGVIILIKKKFGNS*
>group_181
MKKTIFLAGLATVGLLQGTQVKALDATNVTTTPDPQPSAPTNPGVSEQDVAAAQTAYNTATTNAATAQAAYTT
QANAVSTTTTAVNTAKAELTSQQSQVSALEQTASTVSTNVAQTSEQLAAAETAAATAQADATQAQDTYNASVS
EVTAKTAELQAAANQTTTQTETTTVVTTTPATTNDANQPYGKTAPVSYSGDKTEVITLNSSQVTDLTTKGEFT
YTPDYKAVSHFAVDYINEIRALNGIEGRVYYDDTTVEIAIKRAEEMLAAKTLSHFTNLASQYPQYAGDNAAMV
SDSFTYTGLKTLSGVYSDKEYAYKLVLEWFSDHTNVFADDTNNGALNYGHRNALLEHNGGLAVASAHQPLDPT
VPLTQTNRQQLGFSDMPFTVNHQITQWVEIMPGVMEEQTIGDGRVSLDFTYVANADGELVQYLNGKEVVFLPR
TVFEYVYTNTRQVVDAAAVQALNDYKKSAAARLLAEKTAMETTQSISAAKQVSVLALQEKLKQLQSEQATINA
DLSAAKAQVAATTEEFNSLLNKLESEAAQLKALEAANKAAQALRSQLFQTYKDVLAARQATILAEKYASILAE
NKIPVAVVDSTGKIIDYTAQDKPVDTPVDAPVDTPVDAPVDTPVDAPVETPVDTPVDTPVNAPVGFYNNKNTE
SGSKQKIQFVSSKIIDNKFLQNYAGKIELPSTGEDNSVSYAILALSGFSGTLLFIKKRKVQ*
>group_159
MLNFKIHTATCIGNSSNCIYPNEVLVSDRDSFIKAISFDHVCGSFNGSYRSKDNFIKSDCIPMDCDNDHSDDP
DDWVTPFDVALAFPGVCFFASYSRNHMKVKGNKSARPRFHVYFPIEEIKDAGEYSSYKERLYKEFSYFDDNAL
DAARFIYGISNPEVELYDGDLTVIDYLGRRKFEDLPILGSQIQEGSRNSTLSHFAGIILKRYGKSEKAKKAFL
EESEKCNPPLDREELSLIWKSAISFYENISKQKGYIPPDEYKKVSWEKPLPFTGEKMPDFPIEALPKALRNYA
IAVGKSTQTPVDMAAVGVLATVSACMKNLYKVEGKADWHEPTNIYSVIIAEPSERKSAVISLVIKPVDEYIKK
YNQIHKVEFEMSKVIKQRLENKKNSLLSQSKKKGEDKTASEFNDEIRSVVEELVNFTESKPLKVYVDDTTTEK
LTESLAENNNAISIISSEGGIFDVISGTYSSKVNIDVFLKAYSGENISVDRIMRNSIYVENACLSILLSVQPV
VIGELMRNKKFRHRGLTARFLYTTPQSFVGKRTLESECISKDVYREYKELIDNILMEEKTGNTQIIKLSEKAK
ELLKEYFDWVEQKLVGEFTMYSDWLGKLVGNTLRIAGILARSSVIKKDVGDALLEEDSPIVIDEEVFSNAVKI
GKYFLVHAVNAYGDMGVRSDFKAALMVLEKLKEKELVNITRREVMRLCRWVGSAEEAQSILDNLEDYGYIRLS
EIDPAEKMRNGRPKNVVYSINPSVLSE*
>group_610
MFRRSKNNSYDTSQTKQRFSIKKFKFGAASVLIGISFLGGFTQGQFNISTDTVFAAEVISGSAATLNSALVKN
VSGGKAYIDIYDVKNGKIDPLNLIVLNPSNYSANYYIKQGGSTFTSVNQLQTPGTATITYNILDENGNAYTKS
DGQVDIVSLVTTVYDTTELRNNINKVIENANDPKWSDDSRKDVLDKIEVIKNDIDNNPKTQSDIDNKIVEVNE
LEKLLVLPVPDKDKYDPTGGETTVPQGTPVSDKEITDLVKIPDGSQGVPTVIGDRPNTDQPGDYPVTVEVTYP
DGTKDTVIVTVHVTPTPDKDKYDPTGGETTVPQGTPVSDKEITDLVKIPDGSQGVPTVIGDRPNTDQPGDYPV
TVEVTYPDGTKDTVIVTVHVTPTLPTHKCSTSSKKLPGLQNSSKVSKLPATGDEISPFFTSIALTIIASAGLM
LNKKKDN*
>group_0
MRYFDKSQTKQRFTIKKFKFGAASVLIGIAFLSLMGETASAEVISSTSIVADTTSNATNSPDSTALTPTSGQS
TTPSTSTPVESNTSSSSVPISATLVNPTAVEVPPTAPTANALYTDSTLLSGIASAGDTITVTFQNGITATSIV
QPDGSWSLSIPEGMTLNTGDVITIFSTNQKGLKSSLTSVTVIASQSELETPVASPSSGTNSLDLVNNSSISPL
STSSSTAEVQPTALSSNTTTVQPIITSSDISTTQPTASNNVTLKPFNSASVSLSALTTATAGDSSLTVDGKNV
IESAAFNGTTNNTVNGKVVQQWTGNPLEINNPAVATPLAGVRIYAQWVEKSGAVSPIYTTVTMADGTYHIVMQ
DFVLPDGTIVQFDADPNFPEGEKFRIWADTPAGLQLYYSWESGQLGPQSAVMDTSFNASNAIGPNQLNNFNFI
YVSQTQSNVMHNLGDATTSIPAPGETGFINGTVFWNNAVNFGAQTMGASVTNSPGSVDTPATNITVVGSYLSD
YALQQIYTKAATAIGTSNIRGVGWTDANEQALQNWIKQQIAIEGTSLWIGETVTTTTDANGKYNLQFNGTYGK
EWNDRGFNGLLLDSALNSNTVLPDAIAISEGLPIGSKYSDLFNHVSSSPQIGTWYGDASAGTNAMTTDAAPKH

INLDWTYVSLLDVGAYGFSSPYYGNKFLFDTASTSWTNTLKVDAVADQYITGANFGLFLDQLYFDIVNFNTTI
TPASPGNTVTTAVQGLPSPDLGDNKYQIVWFDKNGAEVATSPVQSPSTTGTLPSADFVVPTTAQNGDVYTAKI
FAINPTDDTTRAVYPLAVDSFVVSVTDATTNQPDYIDTTVGAGTSVTIAAPLNTNGTAVPMGTTFTASDPATL
PSWIVVDSSGSLTVSPGVTVSPGIVAVPVTVNYPDGSSEVIDVAITVVDVTAPTITPIANVTTPENKVMTPIT
VQVNDSTATTTVTGLPTGVIYNPTTGQITGTPTIPGTYPITVTSTDTAGNTSIRTFTITVTPDTTVSTAQTVN
PVTSEDTKVTGIGVPGNTITVTFPNGTTGTAVVQPDGTWGVPVPTGVDLVGGEVLPVTSTTPAGDVSPTTNVT
VKDVTPPPAPPVNSVTSEDSVITGTGEPGDTITVKYPDGSIGMTVVKPDGTWTITIPATVDLVGGEVLPVTET
DPAGNVSPVSSVTVKDTTPPAAPTVNPVTSEDTAVTGIGVPGNTITVTFPNGTTGTAVVNPDGTWSVPVPSGV
DLIGGEVLPVTSTDPSGNVSPTTNVTVQDVTPPAAPPVNPVTSEDTVVTGTGVPGDTIIVKFPNGTTGAAIVQ
PDGTWTVTIPPSVNLVGGEVLPVTEIDLAGNVSPATSVMVKDTTPPAAPTVNPVTSEDTTVTGIGVPGNTITV
TFPDGSTGTAVVQPDGTWSVPVPAGVDLVGGEVLPVTSTDPAGNVSPTTNVTVKDVTPPPAPPVNPVTSEDTV
VTGTGVPGDTITVKFPDGSTGTAVVQPDGTWSVPIPAGIDLVGGEVLTATEADPAGNVSPASTVTVTDTTAPN
VPTVNPVTSEDAAVTGVGTPGNTITVTFPDGSTGTAVVQPDGTWSVPVPAGVDLVGGEVLPVTSTDPAGNVSP
TTNVTVKDVTPPPAPPVNPVTSEDTVVTGTGVPGDTITVKFPDGSTGTAVVQPDGTWSVVIPSGVDLKGGEVL
PVVEIDPAGNVSPASSVTVTDTTAPLAPPINTVSSTSTTVTGVGEPGNTVTVTFPDGSTSTAVVQPDGTWSAP
IPAGVTLSGGEIITATQTDPAGNVSPASSTTVSDVTPPTPAVNPVTSEDTVVTGTGTPGDTLTVTFPNGSTAT
TTVQPDGTWTVTIPAGVDLVGGEVLPVTETDPAGNSASVTVTVIDKTPPATPTVDPITSEDSVVTGIGVPGNT
ITVTFPNGITGTAVVNPDGTWSVPVPASEDLVGGEILPVTSTDPSGNVSPTTNVTVKDVTPPPAPPVNPVTSE
DTVVTGTGVPGDTITVKFPTGTTGTAVVQSDGTWTVTIPTGVDLVGGEVLPVTETDLAGNVSGATTVTVTDTT
APDAPTVNPITSEDSVVTGIGVPGNTITVKFPDGTTGTTAVNPDGTWSIPVPAGVDLVGGEVLPVTSTDPSGN
TSPTTNVTVRDVTPPPAPAVNPVTSKDPVVTGKGVPGDTITVKFPDGSTATSVVKSDGTWSVSIPVNVILVGG
EILPVTETDPSGNVSPETTVVVQDVTSADAPTVNPITSEDSVVTGKGVPGNTITVTFPNGTTGTTVVQPNGTW
TVSVPVGIDLVGGEVLPVTSTDPSGNISSTTNVTVKDVTPPLAPPVNPVTSNDNVITGTGVPGDTITVKFPDG
TIGRGDVQPDGTWTVSIPVGIDLVGGEVLPVTETDPSGNVSEATTVTVTDTTAPDAPTVNPITSEDSVVTGKG
VPGNTITVTFPNGTTGTTVVQPNGTWTVSVPVGIDLVGGEVLPVTSTDPAGNTSPTTNVTVKDVTPPPAPPVN
PLTSTDSVVTGTGVPGDTITVKFPNRVTVTTIVQLDGTWTVTIPVSVDLIGGEVLPVIETDPSGNVSPATSVT
VTDTTAPVVDVNPVKLGDTEVTGTSEPGTTITVTLPNGTTVTTVTDHNGNWIVPVSPLQPGETVTAVSTDSSG
NTSQPSSETVPLDTTAPVVDVNPVKLGDTEVTGTSEPGTIITVILPTGTTVTTVTDHNGNWTVPVSPLQPGET
VTAVSTDSSGNTSQPSSATVPLDTTAPLVDVNPVKPGDKVVTGTSEPGTIITVILPTGTTVTTVTDHNGNWTV
PVSPLQPGETVTAVSTDSSGNTSQPSSETVPTMSVENTSATIGQVKTSGNNDKVNVVNNVSQLPKTGEKTNIL
ALLESLLSIATAGLLLMNKKKNK*
>group_28
MNTNNTKGHGYFRKKKWGKGLASGIALAGAVAFSSGAVLADEVTTPASEMETPAVVAQPESMNDNNAYATQAN
TSTGTQTVVIDNSTVEAAAQTATEIGVAVTKEATVDQGTDTNTTDLTASKAEIKADQDQQIKKIEAATQTQAD
NNAAYNEAQAAIDANNDFAEDATAKHEKDTRVTVTTDNSTTTDGSATKNKQAAETAKKVLTTNQTAVQTYIGE
KAVYDATVAQATTLNHAVEAATLELKNKGVTVTTTAKVVSTVAEVKALRKQNEEAIAKANQQVTLNTAILAAY
DKTKQASDATNTDADKKSDELKGSGVTITSSSTEVSSAEEAESIASQNQSAYDQAKQKQADWQKKYDELKAKT
GTEGYTKEVVLQAVDLAAANPQARHGSSAAGATVVGTRDIASSSGRSGYARILDSTGVIKYANVGAGWTTEID
YTNLKGLTVTTADGHKHDISRIHRTFRLVNNGATGLNDVYIPNDPTEGFIVARNNGTDSYSDFMNFLVTDNYY
YTANGKEVAFTATEKTPLALTYSSLNNNQIGREGARATGNGSKMVEINGSTVSVHSDQFAYSNHYNRQEETGV
NWDMTDSPYQYKGAAIGVFTQGEAFTTEFVQWDGPADSGGQTYWFAINTKVVSPVVQVPASATIVKTRVKPVS
VEPVSAQLVKAVNPEKPRLELTKLADTRNQQVSVSYHDYKLSYKPTVTKTVTDTDKKNTDGATVVKNTPQVYT
LNHDNIYGNIKVGDTITIVDPLEAGAVPLVEDNAADAKTKGWTVSYDDTKATYTYTATYQGKKLAAPTIKWTP
IYDKGFFDNTYKVFKNDYEAFSNTVTNYTPEPPKPVKSITDNSGADINGAKTFDRNVNFHVTTDYSPYTTVTA
SSEAILKGFAIMDDVQDGAFTVNEEAITATDIEGKDVKDLFTMYHVLSDDARTQVIQDILDQANLLPVGEFYL
WVAKDPASFYANYVKQAKNVTIDLPARLLVPEGEVVKNDFFQVDFGNSYQSNLVTVEVPDVAPEKHALDQSDD
TIILDDQTVQIGDYIRYLLDGVTVPVKHDTLWQYDGKDKLDVVHDRYTGNWKGIIKGTEYTAKENLVLTYDVT
LEDGTLIKAGDTIKAGSKYAFTFEFDQDTNSDFIKKIVTVTWDEKKGEWAYSIDEEFLRSLGVEGTFDADFYL
EVERIAAGEVENTFVNIDNGKEMTAKVTTHTPEPPQPTPPAPNTPAKPANMLPSTGEASSRLAILGGILLSGF
GLAGVRKRKED*
>group_1597
MKKKQLLSALALSTLVLSQAGFVSADEVTPIDPTAPSTEVVVPPTDTAPVTTDTTSSDSGTTLPVDPTTPSTD
PTTDNSQGKDTAGDDVVAPDTGTQGKDNAGENLDPQPSPEPQGKDTAGDNVDPNTGNTTVPTTDGGTAQVTPD
KTVPTNNPNISADTAHQAGASQVGTTSTVTGQVVQNVSADAPVYTNTGAAIVSTQNGQLVLSDGSTVAPATIG
AVTNADKTITVTQADGTKATLPETGEAHSLLALLGSSLLGVVAWFARKRKMN*
>group_4515
MIPLTPIGGVTIAKENKLPTTGEDENPFFNIAALTIIASVGLLSILKKKED*
>group_4520
MFRRSKNNSYDTSQTKQRFSIKKFKFGAASVLIGISFLGGFTQGQFNISTDTVFAAEVISGSAVTLNTNMTKN
VQNGRAYIDLYDVKNGKIDPLQLITLNSPDLKTQYVIRQGGNYFTQPSELTTVGAASINYTVLETDGSPHMKP

DGQVDIVNVSLTIYNSSALRDKIDEVKKKAEDPKWDEESRDKVLISLDDIKTDIDNNPKTQSDIDNKITEVTN
LEKILVPRIPDADKNDPTGKDQQVNVGETPKAEDSIGNLPDLPKGTTVAFETPVDTATPGDKPAKVVVTYPDG
SKDTVDVTVKVVDPRTDADKNDPTGKDQQVNGKGNKLPATGENATPFFNVAALTIISSVGLLSVSKKKED*
>group_4525
MQDKTLLSEESDIPLLTGQLLKGTLGVGALLGGVSVTQTAFAEQVSSTLPVTSETGSTLAGTDSAVVVASTSS
STSDTSSQSSSLSVSNSQSLSLSTSGSTSLSISQSDSVLQSSSLSQSTSASASTSSANTSTSLAPKSAMTMST
ATTLAATGTVNNNLVTVTSSYIKDTSYNDGYIYPQDGESLEYYTKFTIDNAATAGDQFTMTYSPYTSPSDFDM
ANWTPTDITDSSGEVIATGTWNATTKTVTYTFTTYVDKYQNIVASLDAYSYIDRAKVPNDANLTLTYSLGGET
TSKLEYIRYQSPIVSNTSSIQSVFNELDSVNHTVEQIFYVNPMDYAAYSTYFNVYGYQIDPSTGAMVQQGSTV
INSTTSIQVYKAGTGSTLPDSMDVLDYSTLTKVTPSITYGTDSARIFLGNISDVYVIRVVSTYDPNSANPIVQ
SGTMSSYDYYGYYSIVETNNYVVFTSDTSGGTGTAVTYSIGDYVWLDTNKDGLQTTGENPLANVLVTLTYADG
TSKSVYTDATGHYRFDGLADKTSYTITFTPPTGYLFTTSNVGTNDLVDSDGKTVTVTINGSNNMTVDAGFVPD
TSTSTSLSLSTSVSKSKSTSLSTSTSGSLSTSTSSSLSASTSLSTSISKSTSTSASTSSSLSLSTSSSSSLSA
STSLSTSVSKSTSTSLSNSTASLSASTSTSQSTSTSLSTSASASTSLSTSVSKSTSTSLSNSTASASTSTSIS
NSLSLSTSLSASTSASTSGSLSLSNSISGSTSSSTSLSTSTSASKSTSSSLSASTSGSRSASTSTSTSLSSSN
STSLSVSSSLSSSNSLSQSDSQSISSSVSASNSASTSVVNSASASVSQSISGSQSTSNSASLSTSTSTSLSTS
ASQSASLSQSTSTSRLLSQSVSTSVSESDSTSTSTSLSVSASLSKETSQSVSTSTSVSDSASSSASQSTSILN
SQSLSTSNSQSESQSTSLSLSTSVSSSSSLSQSESTLQSMSISSSTLGSLSDSSSQSESASSSQSLSDSESQS
ASTSLSLSLSESISSSESLSQSEENSQSEIVSTSTLGSLSDSNSQSESVSSSQSTSDSESQSISSSLSLSLSE
SISSSESLSQSEAVSQSESVSTSTLGSLSDSSSQSESVSSSQSTSDSDSQSLSESQSLSLSGSQSGSESLSQS
QSESVSLSLSDSKSASVSESVSLSESLSTSAANSTGVADSASVSDSESRSTSISESISKSQSESDAQSDSESI
SISQSFSLSVTISESESTSFSESLSDSRSLSESTSTSESTSVSDSLSESVNASESLSTSIQDSVSTSVSESIS
VSDSGSLSDSVSQSDSRSLSESTSTSDSTSISDSLSESLMASESMSISEHDSVYTSISESISVSDSGSLSDSV
SLSDSHSLSDSTSVSDSTSNSVSLSNSRKEIDSISASQFILASAIASDSTSVSDSVSTSDSLSQSDSHSLSES
TSTSDSTSVSDSLSASLMVSESLSTNIQESVSTSVSDSISVSESVSISVSILQSESQSLSDSVYQIESLSMSS
SLSDSISSSESMSTSQNQSLSVSVSDSTSVSVSLLESLSLSQNASQSLSSSGSTSTSLVISDSISQAISDSMS
QSDKDSVLLSISISDSTSVSASMSDSSSLSQDNSEYVSNSVSGSGSLSESISTSQLTSVSGSDSLSGSHSASD
SLSQSISHSISASDSVSQSLSQSISTSLSASSSASASTSDSVMVSLSVSGSTSLSTSLSKSTSGSISQSDSQS
ISTLVSQSVSVSVSESLVSSMSISNSLSLSSIISNSLSTSQSSSNKTSQSLSNSQSISQSESDSLSTSLSQSA
AIVGSETAKMYQIGNFVWEDKNANGIQDAGELGIAGVTVTLTKPDNTVVSTVTDSNGYYEFDHLIDGKTYTIS
FETPSGYIPTVADVDPTKDEIDSDGTTVTITINGSDDLTLDSGFVKETYSIGDFVWEDINKNGIQDIGEPGIP
GVTVSLTKPDGTTVSKATDVNGFYQFSHLVDGETYTISFETPQGYSPTISNVDSRDAIDSDGSTVSVTINGAN
ITSIDSGFVKETYTIGDTVWEDSNKDGIQDAGEPGIPGVTVTLTNPDGTTVTTTTDANGHYEFTDLPNGDYTV
TFETPDGYTPTTSNTGDDTKDSDGQVVKVTVAGSDNPTIDSGFVKEVHTIGDTVWEDTNKDGIQDTGELGIPG
VTVTLTNPDGTTVTTTTDANGYYEFTDLPNGDYTVTFETPAGYVPTSSNIGDDSLDSDGTRVTVTVDGSNNST
IDSGFVKVEQPTPGSNSSSESLSQSTTQSSSQSSSAKPVASQTAAQLPHTGQAENNGLYGSAALAILVALGLA
GKKRNEND*
>group_743
MKVKGLLKGAAICAFASTLVACGNGGGSASKGAVEIEYFSQKAEMQSTLKDIIKDFEKANPDIKVKLTSVPDA
GKVLKTRMANNEDPDVINVYPQNMDFQEWAKDGQFLELDNKSGLENLKDGAAKTYAINDKIYSLPLTANAYGI
YYNKDKFKELGIEVPKTFDEFKALVDTINKDGKAAPFALSLNDPWSLNGYHQLAWATNAGGYDGAEEKLIRSG
KDAIKADDAVTVKVNQALELLHGNGQKGYEGAKYTDTVAKFAKGEALMMPQGTWAAPVINQQKPDFEVGMFAF
PGQKEGEELTVGAADLALSISKDSKHPKEAKKFLDYMSSKKAIQKYYDVDGSPTSVKDVDTEGKFKETEGVTK
YAFSDKHIVWLQKEWQSEEEFWNITVKNIKNPSATELAKGLNKFFDSMKK*
>group_4356
LTSGQASADASDKLAHLQALQSLKTKVAAAVEAAKTVGKDDTTGTSDKGGGQGTPAPAPGDTGKDKGDEGSQP
SSGGNIPTNPATTTSTSTDDTTDRNGQLTSGKGALPKTGETTERPAFGFLGVIVVILMGVLGLKRKQREE*
>group_14
MSNQFIQQSQTKKGHGFFRKKKWAKGLASGIALGAVVTFSSSAYADDIANTVDTQPEVTVVSDSNATNLADAQ
ADNSQEHIDLTNQSGEQTGQMTDKVTSDGLDQSVSDAQSAGVEVDQNGTVTHSSYADAQADLAKQKEAIDQAA
ETQKEVDNAKATAEEAAKAAGVEVSTEASKSAKTYTSVEDAKADAKKQVDNLSTVTEAQKQVNEQLPKAVEAA
TDSGVKVNVKAGAKTSDAKKALENLANQVANLKKAQDTQNTISNSMAQALEEAKKAGVTVSTTGSKSYDDLSK
ALADAQAQVAKLTEFKETQASIDEAIKKSADNAKASGVTVSTGDAKTYTSAADAKADSDKQTASLDKAAETQK
AADKVISDLTKEAQNAGLDLTKGTSQTYTSVDEAKKDIAKQEQALKDAATAKANAETTNSSNKKAAADAQAAA
NKAINDAIAKATASGTTVTKTGTIETSVEEAQKIAKEQSDKVNDVAAKNEQIQNENTAKKNAYDAKKGEVDKK
NKAAFDRAGLNYTGDYATDKATADKFNSDEIERVKSQFEKANDNNVLGATDGWVQHVGSNGYTQSGTSQFKKA
QNVNGVEIVANGSLEPGKATVYVKGNVNQSHVIANMNWGNVAPQGDNLTSNIGSKWAEAYDYNADGTTTQIWK
GKILKWYRIPNAITMLDGSQHDAYVLFHTDTSGLSSPGDEVVFWNQDGAINAVDGYRYGVNDKSDGIRTVIRV
DSPDNNDNYLWISLLGDFDIGQFLEADGVTVLGVGGGFATNSGVGGHRVAVDENLGLTYGKNTTPTSALTGFN
SAPDGVALIAQYSNQYSTVIRNTAGGNGVAVARTDFGAQAKVSIVTPRHVNVENVETPTYTELLPVSVKDVHI

TPLQVTFPKAVTLHGVNVQAETHAIKLATEVAPVTVETEVTTPALSATVHDITMKAAVHPVEVTQKPTNEKAV
VNNDNVDINGKTVAKGSTVKWLLKNGNLLAGRKELTAVVMNDPFPAGFEVDSEATAEANADYYTLKTTEDGSY
QLVGTAKLLSLLNAHRDQDVEVPGFVFVGKPLNDNAHYDNAFETIYSTAKGYYKVVSNIPSIDTPDNPKPTKT
VTDKAGSDIDGKTVFDKDVTFHLITDYSTYVNTVVDVNTMTKAACIYDDTDDATVNPDVAKATVTDSNGNDIT
AKGTFYDIEAGQPIPAEIQIFLDGAHLTPNGEFIVWIPNDLKDYYENYVLTGNNVTVNLPVEVIAQPGEKAEN
TFVQVEFGNGYQSNLVFVKVPDVSPEKHAIGKDGTVLDGQEVGLNQVFNYKLDGVTVPKNHDTLWQYDGKDKL
DIEHDRYTGNWKGVITGTEYTAKEDMTLTYDVITEDGTVIKAGDTIAAGTNYHFIFEFNQDTNDDFINKIVKV
TWNAQAGEWAYSINEDFLRSLGLEGTFDADFWIEVERIKDGEVENTFINTVNGRELIAKVTTHTPAAPVPSTP
QVPAKPTAETPVAKASVLPTTGEKTSALDVLASITGLLMIVGAAFGLRKKEKHN*
>group_47
MNKAKVVTAAATVAASFGVVTQVHADDTDLATVSNNTEVAQNQATDNVTKADVDVTKANLDAANQAVSSQEQV
VDDAASTVDSAQEAYNQAQQATSDAQNLADQATPENIANAKTDVNSAQNQVEQADNTEKVAESTAQQAQEAVN
NQSEVVDSAQSDVNTAQSAVDAAQKDVNDKQAILDGTGQAEIIANRDKAQADVNSAQAQVSQKEADLTKAQEA
DANRQIAIDNAKTAVDKANQNVTSTKSDLDAKTATATQTQATEDAAQSAVDAAQKDVNDKQAILDGTGQKAIL
DEADAAKVDKANKETALTEAQKGLKEAQEADANRQATIDNAKAAVDTANQNATSTKSDLDAKTATAQQTEQAL
KDAETAYKTAENDYKAINKITLTQAYVEALKKYNDYSLSSEERQQVLKTLVEESEALAKVNSYKSNPNDDNST
KYQINDLPEDVIKELSQFAADLVNQIRKAFGTPQVSVTSSSVKFADLVTDGYVSDGWNVTKAINEGQVGHDAK
AVNNAARYFGLPTSPSDDEATGGQYYEDWASSAVYDGITFATLKQRVYEAVTDFMFNADEWRHARDIVGGGDV
NSHVYFAIDFSKPSSENGAFAGVHFISVAEDQLTTKNNNFDTNAIANPKSAENVTATYNAAKDSYNQATVANT
AAQSAKSTAQTAYDNAISQLSSASSVLTQAQSVAIQTPTAQANLTSAETALNKAKDRLVKAQKAVEDLNADIK
AKQEALTSAKAVLETAQSTLAQAQSKNSTAQQAKADAQAAYDNAISQLSSASSALTQAQSVAIQTPTAQANLA
SAQSTLTEAKENLVSAQKAVEDLNADIKVKKANLASAKQVLATRQAALATKQNVLKTEQNRLANLQNSLATAQ
DNVSRAQANVVAAKANLDKAKHYLTSLQTAPQLLADAKKQEETARTHLLDALDTLETELLKLKDLQVKQAAAQ
AVYDTTSKAYQTILDAQEKQRLQDEYNAIVAQGKTPVPIVDETGKIVGYQVENIQYSALATEVNNTKATALSV
KTANDTLQKSSEISEATLPETGESSSALVILLGFLMTLWGLVGVRHNKVK*
>group_1647
MKKVKALALFSTAILATASTGVYADEVISDGTTSPSTEQIEPTPAPSSSEVAPGGESNTPTPSSSSSTEAPEG
SKTVPGAAEDESSKRGSETTETEPTQPATEPEKSTETPSEQPGEVEVPTTDGGKATVVPDTSVPTNNPNITAE
TAQNAGASQVGTTSTVTGQVVRDVTRSNPVTLYNGASLVDIRDGLVTLSTGEKVAPEVVGVKANADGTYTAKT
IQGDTVTLPHTGEKNSTVLSVVGALILSVLGFGLKKRKNQEI*
>group_4620
MVVNSIKASNGQDVSSLLEMHHVLSKDALDEKLQAILSNSGLSPIGEFYMWVAKDPSSFFTAYVQKGLDITYN
LSFKVNQTFTEGEIVNGVAQIDFGNGYLGNLVVNDLPKPEVHKDVLDKQDGKSINNGTVKLGDEVTYKLEGWV
VPTGRSYDLFEYKFVDQLQNTHDLYLKDSVVAKVDITLSDGTVVKKGADLAEYTETIYNKETGRYELAFKKEF
LEKVARSSEFGADAFIVVKRIKAGDVYNDYTLYVNGNPVKSNRVVTHTPEDPKPVTPQKLTPKTPALPHTGEA
SVAPLTAIGAIILSVLGLVGFKKRKEN*
>group_4637
VLIQDRSGRYIVSASELGGRLNDDGTVTIKDSEGKEKTLPHTGEEKGFLSIIGGTILSFVAFLFKKKK*
>group_4701
MAILDKEVAKLKELQAVQAEAQNQYSIVFEAFKAVQEAKKQAELTEIYENIIAGGGEAIPAVDETGKITGYVD
GSKKITNETNFNLTSTDKSPANQENKLVSSLTNDLPNTGESSIAPFTAIGAIILSVLGLTSLKKHRTH*
>group_4723
MVAEEIWVGTKPTVETVTIAKPADVEEADATLAKGTRVLKTAGSTGSTTTTTTYSMDATTGVVTANTPTVETV
AASPDVYRVGTKEDVIEIVEEIVEKTKSFGKVVINNPDLLKGISRQVRLGKNGIIEVTYKIVKVNGLEISREI
ISEKEIEKPVDEIVEIGTKEPVENQNQKVIKANESKMNSTDVKKVENKGELPKTGEIKESVFGMFSVIMTGLF
ALVTFLGKRERK*
>group_20
MTTNNTKGHGYFRKKKWAKGLVSGIALAGAVAFSSGAVLADEVTSPVTESEATTVVTQPESTNDNNAYAAQAN
TSTGTQPVAIDNSTVEAAVQTATETGVAVTKEATVNQGTDTNSTDLDASKADIKSDQEKQVKEIKAATQTQAD
NNAAYSEAQGAIDANNDFVEDAKGKHEKDTTVTVTTDGSTDTDGSAAKNKQATEVAKKTLKGNQESVKTYELN
KATYDATVAEAKTLNQAVESAANALKDKGVTVTTTEKVVTSVAEVEALRKQNEASIASAEKQITLNTAILAAY
DKTKKASDATNADADSKVTDLKEKGVTVNVTTTVVSSAEEAETLRKQNEAAATSAGNQVASNNAVLAAYDKTK
KASDATNADADTKAKELQGKGVSVTTAEKEVSSAEEAESIRKQNADAVALAEKQVTSDNAILEAYNKTKKASD
TTNADAEAKVNELKGAGVVVTTSSKEVSSAEEAEKITSQNKSAYDQAKQKQADWQKKYDELQSKTGTEGYTKQ
VVLQALDLSASNPQATHGSSAAGSQIVGTRDIGSTTGSSGYGRILDSTGVFKYANVGKGWTTEIDYTNLKGLT
VTTTDGQKHDISRIHRTFQLVNTGATGLNDVYIPNDPTEGFIVARNNGTGSYSDYMNFLVTDSYYYTVNGKEV
VYSASQETPIALTYSSLNNNPIGREGARATGDGSSMVEINGSTVNVHGDQFAYSDNYNRQEETGLNWDTTDSA
YQYKGAAIGVFTQGSSFTTEFVQWDGPADPAGQTYWFAINTRVVAPVVETPTSARLTKTTVKPAEVKPISASL
IKTTVKPSEVEPVNVSIVKTTVDPVSPDPVSAELVKAKNPEQPTLELTKLADTKNQQVSVAYHDYKLSYKPTV
TKTVTDTDKKNTDGATVPKNTPQVYTLNHDNIYANIKVGDTITIVDPLEVGATPVVDENEADAKAKGWTVSYD

EAKETYTYTAVYQGKKLEAPTIKWVPIYDKAFYDNTYKVFKNDYEAFSNTVTTYTPPTPDPVKSITDNSGADI
NGAKTFDSNINFHLTTDYSPYTKVTASALAIAKGFAILDDVQDGAFTVNEDGITATATDGTDVKDLFTMYHVL
SDEARTEAIQTILDNAKLSPVGEFYLWVAKDPASFYVNYVKQAKNVTIDLPARLLVPEGEVVENDFYQIDFGN
SYQSNLVTVEVPDVNPEKHALDQKDDNIILDDQTVQIGEYIRYLLDGVTVPVKHDNLWQYDGKDKLDVVHDRY
TGNWKGIIKGTEYTAKEDLVLTYDVTLEDGTLIKAGDIIKAGSKYAFTFEFDQDTNSDFIKKIVIVTWDEKKG
EWAYSIDEEFLRSLGVEGTFDADFYLEVERIAAGDVENTFVNIVNGKEMTAKVTTHTPEPPQPTPPAPNTPVK
QANVLPSTGEASSALTVLGGFLISSLGLVGIRKRKEN*
>group_1653
MKKKQLLSALALSTLALAQAGFVSADEVTPVDPSAPSTEVVTPVEPSTPAVTTDTTVPVDVTTTEQTTPVDPT
TPSTEVTPPSDEQGKDNAGDNVDVPEPEPQGKDSAGDNLEVTDPSQGEQPAPSGEQGKDNAGENLDVPSEQPK
TTEQANQQGASQVGTVSTSTGQVVQNVTPDAPVQTNTGVSIVSTQNGQVVLSDGSTVAPEAIGAVTNTDKTIT
VTKADGTKATLPETGEAANFLAVVGTGLLGLVGFLFKKKLV*
>group_4777
VVKVTVDGSDNPTIDSGFVKVEQSTPGSNSSSESLSQSTTQSSSQSSSAKPVASQTAAQLPHTGQAENNGLYG
SAALAILVALGLAGKKRNEND*
>group_1835
MVKKQVAIIGMGVSGLAVLLALSQLPDKLLEKIDITCFDDPKHFGRGIPFQEDSSTAWINSPIDAISYDYHDM
NDFQKWMEQKGLDTDQSYVPRSLYGRYMTERAHDLLQKLKTSVIHEKVTQLNYEPDSQKWNIGTSQKTIPTRF
DEVHLTCGELPVLDPYHLQGNPNYIADPYPLKNLPKQPGKKDRIAIIGTGLAAIDTLKWLLKNSQADLLAFSP
SMTFPTVRILKTETIDWQFLTDTNKQKLFEENSFNCKSLEDLFLSELQALGFQNWEETCRQFLAEGIPGISLS
LAFPAQLFLLQQLASHLVDWLTDFWPQMTLSDRQYYKENYGKAIINLRNPMPEEAGRLLIEATAQGRLQIIEA
VTDIETDDHGFVLKREVGEVLSVATVINATGYHLKESNVHQARTLIQQVIRDGLVQIDPEGGLSILPQTGQVI
SPKYGILATLYAHGSLVNGVIYQNNSTIKIQQMAERAIGNVIKKPTI*
>group_2281
MKAARLLAYNKEKIDLQIEEIAKPIPSRTEVLVEVKYAGVNPVDNMISRGEVKIIVPYKLPITAGNEFSGKVI
AKGTEVTGFDIGDRVYARMPNSKTGAFAEFLTIDQKELAHIPEYLSYEEAASVPLTALTAYQALDLLNVKSGE
SLFISGGSGGFGAMAIPLAKARGLQVITTGSARNAERVLALGADRYIDYQKEDFSQILTSVDHVIDTLGGAEL
EKQFSILKKGGHLVSLKGLPNKAFAKAFGLPKWKQFLLGLVGKKYDRLAEKNHQTYHFLYVTSNGEQLADISR
IFQENKVATSIDSIFTFDQVNQALKKVDKGHSQGKTLLKIH*
>group_2504
MISEQKKRRFLPYLLIGIVMFYFSHWLIKLYAIAPKSSEVTIFDTTAFNWMYDHWSSKPLLDFQFTMFSLYGG
IVVFFIVMLFYFRVTDNGKYRHGEEYGSARYATIKELATFRDKEPDNDMIFSQNARMGLFNKRLPFNKQLNKN
TLTVGLPGDGKTFTFVKPNLMQMNSSFVITDPKGLLVRETGKMLEDNGYKIKVFDLVNLSNSNQFNVFHYMHS
ELDIDRVSEAIIAGTKRSDNNGEDFWNQAEILLMRALIGYLYFDGKVLNKYEPNIAQVADLLRNIRRDDEDIP
SPVERMFEELEKELNGNYACRQWELFNSNFEGKTMTSVLSMISSRFAVFDHDAVRNLISRDSMEMEKWQTEKT
AVFIAIPETDKSYNFIATTMFTMMFRQLPLTADEILQGKHKTCKPGDLLHIRLILDEFANFGRFPNFTETLSS
VRSREISIDIIIQAVSQLKALYKAEWETIFNNCATLIYLGTNDKETMSYFSMRSGKQTLNVKNQSQTRGSQGS
SSQSIQTIQRDLLTPDEIARIGVDEALIFISKQNVFKDKKTNVLQHPRAKELSNSPDDGNWYNYIRSMSGIDD
WDANVNHDRKIIASASEVEDTQLPFQEALAI*
>group_2707
MYHFIPAWYQKDRKWYDDTKEWTHVDQFLQFDDAVNQIRIFKDNQEELEVYVLNYAPHLRTFLHEKDLLEVTV
WSLFDELQGRTQTQASSLNWRHLNWPKGVSFLYSPFTVTALLDDILFARIQFTTDGRVFQILLYDNDSSALKE
SLVFDDRGFLSSIQYFEQGQLRRQDYLDEEGIWRLREHLNQDRGFVIEVNIEKLPFLKQATYANWETLIQEIL
ISKFSNIPANDVLVISADDQHNHFFKEVPAQKVYSFFQDRLPITGGFADNMSFLEGADLLVSDSQKNTEALKS
LDSVETLPAIIQVSPFDTRFELGLSIRRKELEIYFFADQLPDDDIKSSLKIIFKEMAKNPLITLTVGTYQKFG
PRLQWLESLIGDYSDKHIPVEKVSFEDLLPDKNTEEEEPRAKVLVLKAENDVISQFKTSRLVIDLGMQPDLYH
QIAAISSGIPQINQVQSDYVTHQQNGYIISQLSDLPKGIDYFFKGLANWNKSLVYSIEKIADNTGSALISKWQ
SHLKGQDNNE*
>group_2786
MTVNDITLSKEVIQERIARRVAQELENNSLVNLGIGLPTKVAHYIPKDVTITLQSENGFIGLTGLKDKEIDPT
IVNAGGQPIGIAPGGAFFDSSTSFGIIRGGHVAATVLGALQVDKDGNIANYLIPGKMVPGMGGAMDLLVGAKK
VIVAMEHTNKGKAKILNQCTLPLTAKGVVDLIVTELAVMAFRPEGLCVLEIHPDYTFDYVQSVTEVALIDKTQ
EELLA*
>group_2838
MASLKKTIDNKIDYGIITPVFCLILIGLLSIYVATYHDYPQNLSKVMLQQVLWIAFGSLLAFILMFFSTKSLW
KLTPFLYLLGIGLMILPLIFFSPNLVAATGAKNWVTIGSVTIFQPSEFMKISYILALARMTVWYKGKKDRTHF
QDDWKLLGLYLLLTGPVLILLGLQKDLGTAMVFLAILCGVILISGISWWIILPIVLGTLLLILAFFCVFLSPQ
GKTLLYKMGMDAYQMNRISAWLTPFDFSEGIAYQQTQSMISIGSGGFLGKGFNHLDLPVPVRESDMIFTVIAE
NFGFLGAIFLLTLYLTLIYRMLKVTFQFNNLFYTYISTGFVMMILFHIFENIGAAIGILPLTGIPLPFISQGG
SSLISNLIGVGLILSMHYQHILELENESRQQLRRSYKYD*

>group_2886
MSFKDYNFKEYIQKALDAIRFQEPTEVQKKLIPIVNSGRDLVGESKTGSGKTHTFLLPIFEKLKENSQDVQAV
ITAPSRELATQIFEAARQIASYSEEEIRLANYVGGTDKLRQIEKLQVSQPHIVVGTPGRIYDLVQSGHLAIHK
AATFVVDEADMTMDMGFLDTVDKIASTLPKSVQILVFSATIPQKLQPFLKKYLSNPVMEQIKTETVISDTIDN
WLLSTKGRNNNEQILMTLKTMQPYLAMIFVNTKERADELHQFLISNGLKVAKIHGGVPPRERKRIMNQVKKLD
FEYIVATDLAARGIDIEGVSHVINDAIPQDLSFFVHRVGRTGRNGLPGTAITLYQPSDDSDIKELEKMGIAFV
PKVLKDGEFQDSYDRDRRQNREKSYQKLDTEMIGLVKKKKKKVKPAYKKKIQWVVDEKRRKERRAESRAKGRA
ARKAKKQSF*
>group_2903
MNTVEHLIEKFVPENYNIFLDINRDTKTFSGNVAINGEALDHIVSFHQKDLSIQSILLDNEPVIFHEDHKSET
VHVELPVTGLMTLVIEFSGKITDNMTGIYPSYYTVEGVSKEVISTQFESHFAREAFPCIDEPEAKATFDLSIK
FDQKEDEIVLSNMPEINVELRKETGLWTFDTTPKMSSYLLAFALGEVHGITAKSKNGTEVGVFATKAHPSTSL
TFSLDIAVRIIDFYEDYFGVSYPIPQSYHVALPDFSAGAMENWGLITYREIYLLVDSNSTASSRQQVALVVAH
ELAHQWFGNLVTMKWWDDLWLNESFANMMEYVSVDAIEPSWNIFEDFQTGGAPLALKRDATDGVQSVHVAVNH
PDEINTLFDPAIVYAKGSRLMHMLRRWLGDDAFAKGLKTYFERHQYGNTIGRDLWKALSDASGKDVGSFMDSW
LEQPGYPLLTIKVENDQLIISQEQFFIGDHKEHDRIWSVPLNASWTGIPDVLTEKTMVIPNFNQLLSENNGPL
LLNTENTAHYITHYHGQLWESLKSSVSELSEITKLQIIQERRLLAESGHIRYADLIQLMSLFQSEKSYLVTSA
IQQIISGLERFIPENSVEEKNFKTLVSRVFSSDYHRLGFNKQEGESDEDEMIRQIVLNQLIAVDYSDAIGKAN
SLFKDYSNNLADIPAAIRRFVLVNQIKHFETDALVDQYFDLYINTTDNNIRLDLANALSKTQNMMTLKRIIAS
LKDKSIIKPQDLSMWYNVLLSQEFTQNPIWEWARHDWEWIQAALGGDMSFDKFVILPANHFKTEQRLSEYKAF
FEPKLDDMAIRRNIKIGINEIKARVALIEKEKKSRTFNSSKPLMMRRHLV*
>group_3151
MKKSRLAKTSFISLLSVAALSQISVENNHLSFFNVKSVNADEVVEVDATDQGALTDTEVVENATLTSESTVDK
IVSTTVVETPSASFDLTNQNTASIVSEVGSVEVLEAKTSEALSNSVALAATDPIAPDDQTEDVADAKLRVDIG
KVLDDLEAMIASGDIAAAKESYELANTFQGASLEFYGYKDEIDQIPNVKIASVRLEYEARNAAIYQAIYGTPL
PTGPVVTTRTEVIAHTTKYIADDTLAYGQRQTVVAGVDGEKTYTTTDGVEDAGVVTKAMVAEEIRVGTKPTVE
TVTIAKPADVEEADATLAKGTRVLKTAGSTGSTTTTTTYSMDATTGVVTANTPTVETVAASPDVYRVGTKEDV
IEIVEEIVEKTKSFGKVVINNPDLLKGISRQVRLGKNGIIEVTYKIVKVNGLEISREIISEKEIEKPVDEIVE
IGTKEPVENQNQKVIKANESKMNSTDVKKVENKGELPKTGEIKESVFGMFSVIMTGLFALVTFLGKRERK*
>group_3154
VVEVEVPVTVNRDTDGDGIIDSEDPDDDNDGVEDGSDETPKAFTDLTATATPVTVPEGQAVPADTKVVETNKS
DAVISSTPTNGLSVDENGNLVGTPTVDDWGTDEETREVKVPVKVTNTLPDGTEEVVEVEVPVTVNRDTDGDGI
IDSENPDDDNDGVEDGSDETPKAFTDLTATSTPVTVPEGQAVPADTKVNTLPLTGDSSEAFFTSAALAIISSV
GLLGIAKGKKNGSD*
>group_3204
MFKTKKEIFSIRKTALGVGSVLLGVMLTTQVASADEVSLMTPSVDKSLTTTSPVLESTSSQLAATTAPTTTDT
TSNVTATSPVLAAATTAPSVTTTPIASPIRYVSDPNQPVGYRATQVQGTDGSIITTQTGALDANGNPIVTVER
IEPTETVIVLGTKSTSQVTSTQAATTTYSIDVTKPVGTDVVIPAVDGQTTTTTTYKIETATTAPVSPSVLSEG
YKWIDQPFYHVDTTQTLPSDRISIDQLFVPMPVLTPDYNTTESTVREYAQYAENYMYDYITVTNPDGSTTRQQ
VIRPVTSEMLDPTNTRLRTLTGLTDDNAFYSRLFDASQQDLWNTSAQDYGLEIVPEDLSTSTDDFLRYHSSNI
INDALYADIKADYLRAQLAYDQLVSLGTLTSDQQSAMDMMTSQFESLTLRYNNYKDSDAIVVDYSNTTMSATQ
QADFEAKLAALPVEVQRAISELTIYDGQIPGMGETTLGLANSADQTIALKYEANNLNLVSTVLHEMTHIIDFK
SGLYSETTDRNTDGSLSTVMAFSDTQEFLDVYHTYFDRPDVWSYYRDNSEEAFAEGLSQYIMHRLFGTPYSTY
IANPYTGDAYNPGDGSGYSPFAETEFYFASLYNRLFEYPRTAQVVPYLVTTTTTAPVNGQVIYGAMPEETTTT
TPYTTVYVGDTSFAYDPTGQTDRVQAGVDGTETIRTTYSLDSNNQLVATQTVISSTPVQNQIITKGTQPTVVD
TSVPMTIVYQEVTDGSLGDWQVKVLDAGQDGLIRSTTTYSVDPVTGIVTPSTTEATITAMRPMIVQYQVGSEK
VTAIPYQTRYVIDTSLAAGTQVIVQAGVNGSSTESVQSYNFIQDGSNSHFDAIVYSSPVVVAAQDQVIAVGGQ
DQVTDQAVAKTIIYQEVTDGSLGDWQVKVLDAGQDGLVRTTTSYSVDPVTGIVTPSTTEATITAMKPMIVQYQ
VGSPQTSTIPFQTEYVADPNLAYGSEKVVIVGQDGVVTRSVASYDFIQDGVNSRFANITYSEVNNPQVVNEVI
AKGTQTVISDKMIARTVTYQEVTDGSLGDWQVKVVQTGQDGLIRTTTTYSLDSVSGIVTPTSSQSTITAMEPM
TVQYQAGSPKVTPIAYRTRYVADSSIPVGTQAVIQPGKDGTSTIYVDSYQFIQSGATSHFENIVYSNPVVVDA
QDLVIGVGVVNQTHDEIIARTVNYQEITDGSLGDWQVNVLDAGQDGLIRTTTSYTVDSQTGQVSSTRTQNQLT
EMLPMTVQYQIGSSKVTAIPYQTRYVIDNSLAAGTQVIVQEGVNGSSTESVQSYNFIQDGSNSRFDAIVYASP
VVVAAQDQVIAVGGQDQVSDQIVAKTIVYQEVSDGSLGDWQVKVLDGGQDGLVRTTTSYSVDPVTGIVTPSTT
EATINAMKPMIVQYQVGKSKLSAIPFLTEYITDDSLAVGLEKVIQEGVVGTQIETVQSFNFIQDGANSHFENI
VYSSPTIVVAAVDQVIARGTKVDEVVVAVPEVVTPKPETSEVISPEKGQTAPTITVEAIKAPAQKKAKVEAVT
TPKESLPTTGDDQNLLVTLMSSLLLMSLGLGLKKKEDE*
>group_3320
MSITITCQARIMTTFSPKLAIRLNKKYVAEIGNKETIALDIPNNMTLLSYQFLDYPNIKVSNGDHIIIKRNYI
TLTIRWAYILINFFFIYFFKKTLPSTGFINTFLGFSIIPIALLPSYRFIKE*

>group_3370
MRNCKYKLRKLSIGLVSVGTMLMTTTVLGEENTTTTPTTEVTTENQNATSSISNKGDLSNSKTDEVVEETPAP
PGITSTVTTELTETTQKNEKEIAKTINEGGDSVISQQTEDASTTGQTPPQVQTHSRSIRSAEAGQAMEAETME
VEKVSVDKEKTEVTVSDEKKLIKNRDDKQRKIFDVKRDVVVDPDGTTLNVTLTVTPKEIDKGAEVIVLLDTSK
KMTDDDFNTAKENIKKLVTTLTGKDSRNDNKNPNYNNRNTVRLIDFYRKIGEPINLSGLDANKVEEKLNEVRK
KAQEDYNGWGVNLQGAIHRARQAFNSVNEKPSGKRQHIVLFTQGEATQSYEIKEKKDLKKLTVDEPVTYSNPL
LPWPLYFDTTTRKANLVEEGKKIQDYLKKIGIKRYDGLLDSIAQNGNKILNFGSSLVGFNNPLNFITMSDLDT
KNLSEESFDYETKIGEGYHHRSYYDRESGQVPLDSIVKKAVKAKIKKLKKEAEENSYDIFGLSKWSTSLSKVF
GFDTLEEKMVNKFVDYLFYKRNYVYYNHNLSAQAEAKMAREEGINFYVFDVTDPERSSKMDKAWPTGAYGERL
KQKVEEARELSKKRNEKFDKYLKEMAEGKSFFKEVENAEKFKDILTELKVTETFTDKVSVIKDDQLKGEKDVK
HTPASSNFWLFSTSESLIWTISKEKLKDAFEQRTPLELTYKLKVEKDKFKSASPTRAKRSLETPESNKPITEK
AISGKVTYKINGQDGTGKELGDVYVTYSKEKVRKPQEESKVPEAPQLIPLTPLKPSNPIVPAPELPDVEKPEI
ENESIVPDLPEMEQPDQDSPEISGQSQIVDIVEDTLPGVSGQHSSSEETEISEDTRPESDNEIIVGGQSELVD
IVEDTQSGMSGQHSSSEETEISEDTRPESDNEIIVGGQSELVDIVEDTQPSLSGHQSESQETVTVEDTQPNQS
NILIGGQSEIVDIIEDTQAGMTGQYSSTDQLTIVEDTLPEQMEETDEIKADSQVMDIPKVNDTNNDKGAKASV
AFDVEESKVVSTQDIKPSTYVKGDNQLPQTGDDDKVNAFFTLAALSVIGAAGLRQNKRREKERN*
>group_3415
MKNVFRKSKIVETERKSRVKLHKSGKSWVKTTLAFSGLLQLFKGLRVDRELSVSDEDIHLSHHYFIKGIIGIG
GVIGAGALSHTVAADEMVSDLPLASETSATIATLDSAIITSQLDSSQSVSSEQRITSEDNSQTSVVEQSQFAL
GKNSFSERTSVSPTELSESILINTSLAVETNQSDRQTLSESVVYNSISSNNSGSLDNGLVSSESIISSLDSVT
VIESNTLVSDSTLLVSATSETLNNPETTSLTPKNAFKTTQPIILLATGTVDNSLITVTSQSYLESGTQDGIID
PFVMEYLKHTVNFSISNTATAGDQFAYTFGTYVAPDDLNIKQYVPDPITDASGQIIATAAYNQTTKTITYTFT
DYVDKYTDVTASMTVVNLIDRGTVPNSTTVTASSTFASAPAATKSIKVQYSNATKINGTSDLSGMLNYRDKIN
NTIEEIFYVNPLDYISYNYYSSLDIYGYDVNTGISGSDYINSSTIIEVYKVPSTVALPTSMRITDYSLYTKIT
PTISYGTDMATISNLSTFNGETYLVRVVAKYDPASTDPIKISGVMTSYDYYGSGYTATVVNGLKSTSGSSTGT
GTLVTYKIGNYVWQDNDKDGLQGTSSTEKPFANVKVTLTFADGTTKSVFTDAIGYYQFDGLLDGQTYTVSFET
PAGWLPTVSNVGTNDAVDSDGSSITVSINGADNMTLDTGFYQGPSLSASTSTSTSKSTSDSAYSDSVSKSTST
SVVNDSVSKSTSDSVKSDSISKSTSTSIVSESVSRSTSDSVKSDSISKSTSDSVVSDSISKSTSDSVISDSIS
KSISLSQESDSVSRSTSNSQLSDSLSQSTSLFQESDSVSRSTSNSQLSDSLSQSTSLSQKSDSVSRSTSLSQE
SDSISKSTSLSQESDSVSRSTSASQESDSLSQSTSLSQESDSISQSTSESAYSDSVSQSISMSEESDSISQST
SESAYSDSVSQSISLSEESGSISQSTSESAQSDSVSRSISVTEESDSISQSNSTSAQSESLSQSTSMSQESDS
LSQSNSTVAQSESLSQSTSMSQESDSLSQSTSDSAESDSLSQSTSLSEESNSLSQSTSQAEESDSISRSTSMS
AESDSLSQSTSDSAESDSLSQSTSMSLESDSISRSTSESIASDSNSYTSQQSDSLSQSISLIQESDSLSRSLS
ASEESDSLSQSTSLVEENDSTSRSTSASVESDSLSQSVSQIEESISLSQSTSASVESDSLSQSTSIAEESDST
SRSTSVSVESDSLSQSTSIVEESDSQSRSASASVESDSLSQSTSIVEESDSQSRSTSVSIESDFLSQSTSVKQ
GSESLSDSKSLSQESDSVSQSTSVKQESESLSDSRSLSQESDSVSQSTSVKQESESLSDSRSLSQESDSVSQS
ASVKQESESLSDSRSLSQESDSVSQSTSEKQESESLSDSKSLSQESDSVSQSTSEKQESESISDSVSLAKESD
SVSQSLSNELEYESISSSESLSQESSSISESLSITVHSESVSQSESLSQESESISESLYRVSNSISVSNSDSS
SLSTSISQSQSTIGSEAPKVYAIGDFVWEDTNKDGIQDAGEPGIPGVTVTLKNPDGSTKTTTTTDSNGYYEFT
DLNDGDTYTVTFETPKGYKPTVANIGDDSKDSDGASVTVTINGANDITLDSGFVRDLHTIGDTVWEDTNENGI
HDASEPGIPDVIVTLTNPDGTTVTTSTDANGHYEFTDLPDGDYTVTFETPKGYNPTTPNTGDDTKDSDGQVVK
VTVAGSDNPTIDSGFVKKAYTIGDYVWEDTNKNGIQDAGEPGIPGVTVTLTYPIGSTVTTTTDANGYYEFKNL
NDGETYIVTFETPKGYAPTIANTGDDAKDSDGSSVSVTIKGADDITLDSGFVKVHTIGDTVWEDSNKDGVQDS
GEPGIPGVTVTLTNPDGSTETTTTDEKGHYEFTDLPDGDYTVTFETPDGYTPTTPNTGDDTKDSDGQVVKVTV
EGNDNPTIDSGFVKETYTIGDYVWEDTSKDGIQDAGEPGIPGVNVTITYPDGSTKTTSTDSNGYYEFTELNDG
DTYTVTFETPDGYKPTVSNNDDDSKDSDGATVTVTINGADDLTLDSGFVKDLHTIGDTVWEDTDKDGTQDAGE
PGIPGVTVILTNPDGSTETTTTDENGHYEFTDLPNGDYTVTFETPNGYTPTTSNTGDDTKDSDGQVVKVTVDG
SDNPTIDSGFVKEVHTIGDTVWEDTNKDGIQDAGEPGIPGVTVTLTNPDGTTVTTTTDANGHYEFTDLPNGDY
TVTFETPDGYTPTTSNTGDDTKDSDGQVVKVTVDGSDNPTIDSGFVKVEQPTPGSNSSSESLSQSTTQSSSQS
SSAKPVASQTAAQLPHTGQAENNGLYGSAALAILAALGLAGKKRNEND*
>group_3432
METKEIYSFRKFKTGTHSALIGKFGIALTTSIAIMTAGGVVHANTITTSTTSQVTPTTSVASAEIPTSVPAET
SQSTAEKTINSAQDTLRTNVSNAAKAGVDVTTGTTTDIILNNSNVVDKINEVLTDLSHQDKAVADAKAKQEVN
QKAYTDAKTTRDTAVSKGQSDLSHAEQGVDDQITIAKKDSISVTTDSKDLTPKYIDTKGLTGSALTTAMEKNI
ALYNQAVNDGVGIMDASSVQMKKQIADYLTALTNYQKGVSSNTGLQWQNSVVLEAGSGASRQTGTENVVDFSD
GTIKAAGMYATQGQNLDQNTDANFDNIFKIEGTGIIWVKNTTNGDVKLTFSEINSPYNTGTYVAIWGDDKGGI
AWSVFALYYGGASGGAGETGSNTGSGISGRILNYVNSYKATVETTRGVSVVTFNDIDNQQTVKMSGLDNAKVT
TGENINQTGNDFVAGSGDVSQSSAGVLGTNGVKWTFTSADRRLFSFTHSTAGTKNTSIVGGIFGSASNVPQKP
VIPKLTAHKAIVTGPKGPSIPVNQKVTVHYYKVSVTPTPENPVTPQKNITYKPITTTPQTPVSQANVGLPTTG
DKADGSIVQMVIGALMVSFVGFSALKDRKKEK*

>group_3433
MRETKGHGFFRKSKAYGLVCGIALAGVFAFNGGNVSADEVTAPATQPQTVVTTLTEPTNNQAQSVTSTALDTA
VSTAKEAGVTVNTTSTVSHTDVSSALSDLANQTKAVNDATAKAEANTQAIKDATAQNAQIDAQNQAEATRVAE
ANKAGQLAVDQKNAQGQALVDKQNADAQAQADATNAQLKADYEAKLAEIKTVEDYNKAVVERNALAKQQADAT
NAQAKAEYDRLLSIYNKALADRVNLVANDITFEGYGKSEVLDSYGQKTTDVVTVNNDGTFVLTEPQSDTDGTF
GNTKVTGKFIYTVAFDPATNQAVIKITGISLNTWQLDLTRQTTAVNRNISASYKDLAGNVLYTRHFDGLSSIG
VQTINKSFTLDKEIILSNGQTSEEVMFLKTVPSWIYEAPSSLYAKLSYNTNALPPKPQEPTLVTVTPEALKPV
PQTPPVPVYVKPVLKTFTPEVYTPIKPTVKPHVAVPDKLVVTVSVHPVVVPVANPAKEVVDQAGKSINGLSVL
PNSDLNYVTKQDFSQYKGMTASNDKVAKNFIYIDDYDEKSLDGKSMVVNSIKASNGQDVSSLLEMHHVLSKDV
LDEKLQAILHNSGLSPVGEFYMWVAKDPSSFFTAYVQKGLDITYNLSFKVNQTFTEGEIVNGVAQIDFGNGYL
GNLVVNDLPKPEVHKDVLDKQDGKSINNGTVKLGDEVTYKLEGWVVPTGRSYDLFEYKFVDQLQNTHDLFLKD
SVVAKVDITLSDGTVITKGSDLAQYTETVYNKETGLYELAFKKDFLTKVVRSSEFGADAFLVVKRVKAGDVYN
DYTLYVNGNLVKSNRVVTHTPEDPKPVTPQKLTPKTPALPHTGEASVAPLTAIGAIILSVLGLVGFKKRKEN*
>group_3633
MKKKQEMQYYLRKSAYGLAAVSVAVLAVGSPVSAQEKAASTEATPKVGPKVPKKPDRELLKKLLEEAKAKEET
AKKAKEKLIKPAALTDEESLVITEKAEELSDKVLGEALSKEEAEPSEEENKTDEEESDGVESGEIEKTESEES
TESEGVKEESEEEKEDDPSESETEVENVEAINLSEAEGNDSSKPETSEEVTAEEDRQETDRLAEVKTEESAKE
GDEDADKKDEVEEKAKKAAELSRVKAEALAKLEALNARRLMKKIVESGKTVEGILSFMKESLPQLEAARASEQ
AKAPEVTQSPDHLPSEKKAVHNPVQAAKRSESLEQKAENATTSTNLQNTQSPVQEAKRTQAQLPSTGEDYQAY
LVAAAMALIASSGMVAYGSYRKKKQK*
>group_3639
MSKEVLERRVEVSANLDDYVYDYEKKHANFLKSGDSNDVAKQNAVQDLKTNLTKDNLTLVDTTYDPDTGVAAI
AVKDSQTGETYISYAGTNLDADGLKDVGSDLSIGLNDPIHLKKLGNQATAFYDQIQATNGTNITVVSGHSYGD
FLATRVAIERQVPFKFGFQGAPQSVSKLTAQEIVVNQIYREGGIPPKELLDAVDSARVESDRVSKLMENYKGY
AVTFSTTGDLLTNGLWRQSDREINFDGSKINGPSTWGLGFLSGTLGTFPDTKYVGHVIAIDVNTEHSMYNYRV
DQSVMAYTKQIISENILGVDFNQDGNQEFFLTQDYLTTQSLLPMTAQSAKEIKLDKKAMTVLSNNLRNVKSQI
ESLIDLTSQAKRSNDDVLSRLSSRRQSLKDAIMRELEAVSLIEAVRQIDDAFNELGNLENDISTVANYDSYSF
TRKFDWFGDSGNYEWYQDGSKWDYGPISRLLSDSSWEASCLKSLISDNQLTCTPGYGYRYNTNFCAYPAKTDV
AGEGVELINSFEKMIEETTSGLDNRSHFDDGIPQATKEILQVVLDNLTTLKGCTQYLISVTELIHRGLSDSDD
SLATDIKNLDLSRVPEVRVTISDDYQTFLEESKVFDDKTVLVAFDDQVDQKSEDLADKMSKAYGEYLSTVEHQ
IESVNRYLGKLAENFSQLSHDMPKQLQYKKEEFHLFSEDNDQTPLMDYGKIGDHITVSSTISKLAGNGAGTGI
EQVDTKLTQAATTINTVYSYLGNFKQPFRLGMEEAFYGVSGLDEIVKSQLAVSSVLKAMHQRFKEFQSTLSYH
SGLAVEALSDKLDGTLLTLSNVSKMLDECFGD*
>group_3645
MFKKTNNKYAIRKLNNGANSVVIGAIGMMALCGGFARTVSAEQVTTNTTQTQPITVVSPTNQLSPAIALAATQ
PAVVVSNPDAEIVTTTTPAPNPTAPAKTEDKMVTVTEPADQVNQVVEGGTHTETSTRRGDTYSYNAQGSTTKT
IVQTGVDGTTTVTYRVVYDYNGKEIARDVISVTQNPTEKPEINNYTPDIIVDEHEVFNHYLPGDTPYTGGIHK
PMFVPLSVPFESGVALKLDDIVWTDGITKEFAQNMVFTFNTWDEFISKNQSVPGFELFLNFLLAQEKYRNAFT
DTSHTTLPNYRWDPNTGEQITDPSDIQEIVNRSTNGNNAPDAEQVDLTNYSLKDKIALLNEKFYTAPVYYPEG
TLITNPDGSPVFDEFGNPTFYPANTPMLKEDGTPLTVRDRLVQSFQYYLNIREYWKAHEDIKRDVIVRFNPNL
KSSEKVLVQQGQDGRIEYAHFDYYAKYIDGPAQYYFGPGPMVAMSIEPATSTPISTSTTDINGITYVTDPMTG
EKYIDANSYYLLSEINKNVAQKNFSYSSMQVGDIYKKTLPIYVGTQVNNPNYDPSQPESSTNLRFIIEYSIQE
MPLDVIITQFVQATPDIYEYGVDAFTSIATVLPRTDLALTDTETLEVPFNTEVRYNPNLKPGESRIVQVGSNG
TTEVTVTYDSATDTYPTDIIETVISTPVSEIIEVGTGVTGQAVEHSLEVIDYAIEIVYDSNLTSGTVQIDQKG
VNGYKDVLTTTETLNGQVIGTAKTTTNQKDPLNTIIRIGTKIEPTPEKNVVVTHEESVQPQIIEYRYNDQLTA
GTSNIIQKGQDGFTVKIISQPTVNGIPQGDPTVQLVTQDAVNTIIELGTRPIEVIKEVPVEVIKEVPVEVIVE
KEVIKEVPVEVIKEVPVEVIKEITVEVIKEVPVNVPVDKIVEVVKEVIVEVTKEVPVEVIKEVPVEVIVEKEV
PVETSITVETIKEKIAYPTEVRKNPDLSAGEIHVIQKGIFGESAVTTKTTVVTKNGQSTSTKTEDISTIIDPV
AEVIEVGTKVDIPVIPPIEHETPTDPVNPTEPTVPLTPPIEPIVPSVPDSPTPAPTVANATALASYQVAPVKK
EGVLPVTGSHDQTALLSLTSLSSLALGFALLQTPKKKED*
>group_3657
MVQIVLEFFLGNISDVYVIRVVSTYDPNSANPIVQSGTMSSYDYYGYYSIVETNNYVVFTSDTSGGTGTAVTY
SIGDYVWLDTNKDGLQTTGENPLANVLVTLTYADGTSKSVYTDATGHYRFDGLADKTSYTITFTPPTGYLFTT
SNVGTNDLVDSDGKTVTVTINGSNNMTVDAGFVPDTSTSTSLSLSTSVSKSKSTSLSTSTSGSLSTSTSSSLS
ASTSLSTSVSKSKSTSLSTSTSGSLSTSTSSSLSASTSLSTSISKSTSTSASTSSSLSLSTSSSSSLSASTSL
STSVSKSTSTSLSNSTASLSASTSTSQSTSTSLSTSASASTSLSTSVSKSTSTSLSNSTASASTSTSISNSLS
LSTSLSASTSASTSGSLSLSNSISGSTSSSTSLSTSTSASKSTSSSLSASTSGSRSASTSTSTSLSSSNSTSL
SVSSSLSSSNSLSQSDSQSISSSVSASNSASTSVVNSASASVSQSISGSQSTSNSASLSTSTSTSLSTSASQS
ASLSQSTSTSRLLSQSVSTSVSESDSTSTSTSLSVSASLSKETSQSVSTSTSVSDSASSSASQSTSILNSQSL
STSNSQSESQSTSLSMSTSVSSSSSLSQSESTLQSMSISSSTLGSLSDSSSQSESASSSQSLSDSESQSASTS

LSLSLSESISSSESLSQSEENSQSESVSTSTLGSLSDSSSQSESVSSSQSTSDSESQSISSSLSLSLSESISS
SESLSQSEAVSQSESVSTSTLGSLSDSSSQSESVSSSRSTSDSDSQSLSESQSLSLSGSQSGSESLSQSQSES
VSLSLSDSKSASVSESVSLSESLSTSAANSTGVADSASVSDSESRSTSISESISKSQSESDAQSDSESISISQ
SFSLSVTISESESTSLSESLSDSRSLSESTSTSDSTSVSDSLSESVNASESMSTSIQDSVSTSVSESISVSDS
GSLSDSVSQSDSRSLSESTSTSESTSISDSLSESLMTSESMSTSEHDSVSTSVSESISVSDSGSLSDSVSQSD
SRSLSESTSTSDSTSVSDSLSESLMTSESMSTSEHDSVSTSISESISVSDSGSLSDSVSLSDSHSLSDSTSVS
DSTSNSVSLSNSRKEIDSISASQFILASAIASDSTSVSDSVSTSDSLSQSDSHSLSESTSTSDSTSVSDFLSA
SLMVSESLSTNIQESVSTSVSNSISVSDSGSISVSILQSESQSLSDSAYQIESLSMSSSLSGSISSSESLSTS
QNQSLSVSVSDSTSVSASLLESLSFSQNASQSLSSSESTSTSLVISDSISQAISDSMSQSDKDSVLISTSISD
STSDSVSMSDSSSLSQANSEYVSNSVSGSGSLSESISTSQLTSVSGSISLSGSHSASDSLSQSLSLSISASDS
VSQSLSQSISASLSASSSASASTSDSVMVSLSKSGSTSLSTSLSKSTSGSISQSDSQSISTLVSQSASVSVSE
SLVSSMSISDSLSLSSIISNSLSTSQSSSNKTSQSLSNSQSISQSESDSLSTSLSQSAAIVGSETAKMYQIGN
FVWEDKNANGIQDAGELGIAGVTVTLTKPDNTVVSTVTDSNGYYEFDHLIDGKTYTISFETPSGYIPTVADVD
PTKDEIDSDGTTVTITINGSDDLTLDSGFVKETYSIGDFVWEDINKNGIQDIGEPGIPGVTVTLTNPDGTTVS
KTTDVNGFYQFSHLVDGETYTISFETPQGYSPTISNVDSRDAIDSDGSTVSVTINGANITSIDSGFVKETYTI
GDTVWEDTNKDGIQDAGEPGIPGVTVTLTNPDGTTVTTTTDANGHYEFTDLPNGDYTVTFETPAGYVPTTSNT
GDDSLDSDGTRVTVTVDGSNNSTIDSGFIKETYIIGDTVWEDTNKDGIQDAGEPGIPGVTVTLTNPDGTTVTT
TTDANGHYEFTDLPNGDYTVTFETPNGYTPTTSNTGDDTKDSDGQVVKVTVDGSDNPTIDSGFVKETYTIGDT
VWEDTNKDGIQDAGEPGIPGVTVTLTNPDGTTVTTTTDANGHYEFTDLPVGDYTVDFETPAGYVPTSSNTGDD
SLDSDGTRVTVTVDGSNNSTIDSGFVKETYTIGDKVWEDTNKDGIQDTGELGIPGVTVTLTNPDGTTVTTTTD
ANGYYEFTDLPNGDYTVTFETPAGYVPTSSNIGDDSLDSDGTRVTVTVDGSNNSTIDSGFVKVEQPTPGSNSS
SESLSQSTTQSSSQSSSAKTVVSQTAAQLPQTGQAENNGLYGSAALAILAALGLAGKKRVEK*
>group_3674
MSKPMTKKKKAISIQKSVKPILGFTFGALLLSTVFTPSVFAEEVASSLGHATSGLLSVSVPKELTSLETTTYL
MASESPSNTLTSDTISSDNGGTASNPNETVTTETTSEAIPFDTEVIQNPDLPIGEIKVVQEGVAGEVTVTKTT
TTITQNGVSQSTTTESRVPVKKPINKIIEVGTKEISTSPSSSDVITVSPSPSSTSSESNQQGSLTPAPKSRQN
SQEKKGSQTKKSKDDAKEKEGDKKELPPTGSQESGIFSLFSALISTALGLFLLKSNKND*
>group_3695
MKKEKLLSLITISGIALLGSTSVFASDVTDTLIDNQPVVVTTSPSDPVSDTTDTSTPIPSPSDPVSGTTDTST
PIPSPSDPVSGTTDSSVPIPNTDDTANNDSSTATSDTNIEKPLDKPNENIPTDPSKPVPIQEEPVKPVTKPII
DAPITTETGAQIVGTQDGKVLVQTETGTQVKEAKEVGGEVQKDGTVVIKKADGKIEVLPHTGDSKKVFTVLGI
ILILGAFWVGFKENIKKFLTMFSKKEKIRVGKAMINFMAFFFLDNI*
>group_3850
MEIKQKHGKHALRKAVTAAVLAGTAFSSLGGFAGNLTSVAADQIVKGRVFRPNLVDTQDYEKIISAFASFRAK
EGSVADLRKVLKDASLDTLYILLSGLDPADLTYNGESNILQTLAKMLNTFENDKEENEDFYHRRGINFITLLI
QEITDRIVQNEQRGKEIIKKDSELILMNDKVKTLESDKENIENKLQDKSQELAEKEEKLKETEKELSESRHDF
ADVNEELEKVKETVKEKEASVKDLTEKLETSKKNHAELAEEFEKTKGEYEKELSENDKKLSEVAKENSDLVSE
NKELKENLGTAEEITNDLQKKVMNAEKAMKELEEELKAEKEALEAEKAKLAESEKANEKLTEERDAAKKEAEK
VPELEEQVEKLVEEITAAKKEAEDLQAKAEALEKDFEAVKAEKEKLEAEIAKMKEDHQKEVDALNALLADKET
MLKNLQDQLDKAKEEAMKNEQMSQEEKAKLQAELDKAKQELADKIKDMPNKVAPQAEGKANAGQAAPNQNQNN
QAQANQAKNGNNLPSTGDKPVNPLLVASGLSLMIGAGAFVYAGKRKKG*
>group_3863
MLKKQLLVLTCVSTLGLVGTTAFAEDVVPVDPTTPSTEVITPSTPIDTGLPSDTTDPSTPVEPETPTNPTNPS
TPIDPGTPTDTTTPTDDTTTPGTSTSPSDSESTNTNPGTKPTDEVKPVTPSTPEVPSTVTPGVVDNVDKETGN
ITIKPIPISPDKTVVGTQNGNVLIQDRSGRYLVSASELGGRLNDDGTVTIKDSEGKEKTLPHTGEEKGFLSII
GGTILSFVAFLFKKKNNFKLTKKQ*
>group_3895
MTHMNKNGRYKQRFSIRKYKFGAASVLLGTIFALGMTGTTAQAQIYNDYGGYYGAPEIYSRGFNGGLATFPGV
GSNPTYSNVDGYPISNVNPNLNNYGSYGYQPSTYSTASYYDSNYNNYNLLQGPPGIPGPPGTPGIHGMNGASG
LAGPAGPQGPSGAPGAPGPVGPKGEPGQVGPSGPAGPSGPAGKPGEPGPAGPQGEAGPKGEKGDPGKKGEKGD
RGKKGEKGDRGKKGEKGDRGPEGRKGDRGARGRDGQKGAKGDSGRDGRDGQQGPEGKPGRDGHNGAPGRDGLN
GEKGDPGKNGLNGKPGAKGADGQDGRDGRDGNPGKPGKNGAKGAPGSDGAKGRDGQDGAKGRDGKDGTNGVAG
KNGRDGAKGRDGKDGTKGQDGKHGRNGQDGRHGQDGVDGRNGLDGRRGRDGRDGRDGIDGVDGRDGLSPIIKT
MTHSDGSHTIHFLNPDGTRSEITLRDGKNGQDGKDGAPGRDGVDGKDGAPGRDGRDGKDGMPGRDGRDGHDGK
DGMPGRDGMNGKDGQAAAGNTAGKANASDMKPKAMAAPAAMTNQNAHANNNGPAKAQLPSTGDKANPFFTAAA
LAVMASAGMVAVSRKRKED*
>tolA
MTKTCNHHFLVNQEKGEKHVFRKSKKYRTLCSVALGTMVTAIVAWGGTVAHADEVTPKVDTTIQRTENPATNL
PEAQPTPVSEQTESLVSTGQSNGGIAVTVPHDTVTQAVEEAKAEGVSTVEDSPMDLGNTTSASETSQQISKAE
ADAQKQVEAINEVTESYKADKATYESNKARIEQENNELSQAYEGANQTGKETNAWVDTKVNDLKNQYADADVA

VKEQVVSSGNGTSVLDYTNYGKAVETIQSTNEQAVADYLTKKTKADEIVAKNQAIQKENEAGLTKAKADNEAI
ERRNQAGQAAVDAENRAGQAAVDQANQEKQQLVSDRAAEIEAITKRNQEKEAAARKENEVIDAYNTKEMERYQ
RDLAEISKGEEGYISEALAQALNLNNGEPQAQHGAITRNPNQIISTGDAMLGGYSRILDSTGFFVYDSFKTGE
TLSFNYQNLQNARFDGKKISRVTYDITNLVSPAGTDAVKLVVPNDPTEGFIAYRNDGNGDWRTDKMEFRVVAK
YFLEDGTQVTFSKEKPGVFTHSSLNHNDIGLEYVKDTSGKFVAINGSTVQVTNEGLARSLGFNRASDLNLPEE
WDTTSSRYAYKGAIVSTVTSGNTYTVTFGQGDMPQNVGLSYWFALNTLPVARTVTPYSPKPHVAVELEPVPEP
ITVTPDVFTPKTFTPEKPVTFTPKPLEEVVQPSLTLTKVNLPVKPIPKELPTPPQVPTVHYHAYRLTTTPEIM
KEVVNSDQANLHEKTVAKDSTVIYPLTVDALSPNRAQTTSLIFEDYLPAGYLFDKETTQKENGNYVLSFDATK
NFVTLTAKENLLQEVNKDLTKVYQLNAPKLYGSVQNDGATYSNSYKLLLNKGTTNAYTVTSNVVTVRTPGDGE
TTTLITPDKNNENADSVLINDTVVALGTTNHYRLTWDLDQYKGDRSAKETIARGFFFVDDYPEEVLDVVENGT
AVTILDGQKVSGITVKTYASLNDAPKDLQYKLARAKITPTGAFQVFMPDDNQAFYDQYVKTGTSLALLTKMTV
KDSLYGQTKTYTNKAYQVDFGNGYETKEVTNTLVSPEPKKQNLNKDKVDINGKPMLVGSQNYYTLSWDLDQYR
GIKADNSQIAQGFYFVDDYPEEALLPDEAAIQFITSDGKTVSGIMVKSYAQLSEAPNTLQAALSKQKIQPKGA
FQVFIPEDPQAFYESYVTKGENITIVTPMTVLETMLNSGKSYENVAYQVDFGQAYETNTVTNFVPKVTPHKSN
TNQEGISIDGKTVLPNTVNYYKIVLDYSQYKDMVVTDGVLAKGFYMVDDYPEEALTLNPDDIQVLDKDGNRVS
GISVSTYASLSEAPKVVQDAMAKRQFIPKGAIQVLSSDDPKTFYEIYVKTGQILVVTLPMTVKNELTKTGGQY
KNTAYQIDFGLADVTETVVNNVPKLDPQKDVVIDLSHKDESLDGKEVALHQTFNYRLVGAMIPSNRATDLFEY
GFEDNYDEKHDEYNGVYRSYLMTDVTLKDGSVLKEGTEVTKYTLQQVDTENGLVSISFDKSFLETISDDSAFQ
ADVYLQMKRIAIGQVENTYLHTVNDYVISSNTVVTHTPKPEEPSPNQPTPPQPPIETIEPPVPSSVLPNTGEQ
ESLWGLIGAGILLGTAYGLKKKEER*
>group_3961
MKIKIKNNMRNNIKKNHRQPRYALRKMSVGFTSCVIGWCIFSSSTTVHASVLPDTSISTSTTSINNTTQTSPI
TENVVDTSIITKPIDSNKLIESTATTIETTSTVETITVALENNTAVEPTNTTVENTTAVKPSTATLETTTAIE
PITTSVENTTAVEPITTSVENTTAVEPITTSVENTTAVEPITTSVETISSTDGIATNSTSTSTSQLQPKMLMM
ISPMSVSPTAPSIYISNMTELLNAINNYSDVNIFIKGTTYLIDKAISIASGKNISFNVDPSSTEPVIFLRDVS
KGYLSGSLVTVEQGASITFNSDPGQMIIDGGSTDNPSSGIVSETGLLLTNKGETIINGAEFRNQNLFYGSYIA
PIYTSGPGSKLVINSGSIHDNKITANSERIIYSSTAILAENESQVFLNGGEIYNNTIDKSLINSDSVGVINIS
DGSYFEMNGGQIHHNSARSGVTIGDSRFYAYYIKPETWQTEGTIKNNPTGLLTIDQIFVPGKRMPKIAKAVIN
NGTINNNMAYANGGGLNVWSASEVTINGGKIENNETNGYGGGISVIDNYLNYLGGYTRKIANVKRSEWEQYLG
AKLTINNATINGNKSTDGRGYGGGMFIASNLVTINKADISNNMAVNGASIAMTDKAYTLKLKNVSTDQSIGNS
SFSNSSDSNLYIYEKDGLVFTPINPGTGTSNSNYDLTANWATNNILDPAYRMLGGGLINYIDKLGNTVNAEPK
YSNSVTPTTPAHYIDPIDGYATKSSLDYGKTLASITMNGNYSVYYYRALNRYYPSYSSGSNFFNGGTLIIGTP
DNIKNEIDVNATLNFKTIYDSFLAQDPNNILKLTVNYETPMGNVILEEHDIPSLEGLVQIIGNLSETNDLSKL
KFQFSGINTDETITLTADNFIKSETISSTERVWNSDTFMFDEKTYYTTLNHFSILLDTVIKSVETITSLPEKI
IYDSTKPIGTEIVEPGVDGLTQTNSTILKINGIESDVIMTSQNVIKEPIAQIRIIGTGVVGQNVDVTFEVIPI
PTQPTIIEDETIPLGEEVIVETGSEGQIKVTTTTPTLNGIPNGEPIVTREIVTQAKAKVIKRGTGISSSNIDT
IKARITKAISDKKHEVNANETIILPNTGFKHNHKQTYGLLSILLSLILFVFKSKRRTK*
>group_3392
MSASTSASTSASTSTSVSASTSTSTSASTSASMSASRSASTSASTSTSVSASASASTSASTSASTSASTSAST
SASTSASESASTSASESASTSASTSASTSGSESASLSASTSASTSASESASTSASTSASTSASESASTSASTS
ASTSASESASLSASKASLPNTGETDDSSSFITSLLALLLALGLISKKRDEK*
>group_4004
MFKKLNHQTVFETEAKSRVKLHKSGKSWVKTTLASIGLIHLCKGMQDKTLLSEESDIPLLTGQLLKGTLGVGA
LLGGVSVTQTAFAEQVSSTLPVTSETGSTLAGTDSAVVVASTSSSTSDTSSQSSSLSVSNSQSLSLSTSGSTS
LSISQSDSVLQSSSLSQSTSASASTSSANTSTSLAPKSAMTMSTATTLAATGTVNNNLVTVTSSYIKDTSYND
GYIYPQAGESLEYYTKFTIDNAATAGDQFTMTYSPYTSPSDFDMANWTPTDITDSSGEVIATGTWNATTKTVT
FTFTTYVDKYQNIVASLDAYSYIDRAKVPNDANLTLTYSLGGETTSKLEYIRYQSPIVSNTSSIQSVFNELDS
VNHTVEQIFYVNPMDYAAYSTYFNVYGYQIDPSTGAMVQQGSTVINSTTSIQVYKAGTGSTLPDSMDVLDYST
LTKVTPSITYGTDSARIFLGNISDVYVIRVVSTYDPNSANPIVQSGTMSSYDYYGYYSIVETNNYVVFTSDTS
GGTGTAVTYSIGDYVWLDTNKDGLQTTGETPLANVLVTLTYADGTSKSVYTDATGHYRFDGLADKTSYTITFT
PPTGYLFTTSNVGTNDLVDSDGKTVTVTINGSNNMTVDAGFVPDTSTSTSLSLSTSVSKSMSTSLSTSTSGSL
STSTSSSLSGSTSLSTSVSKSMSTSLSTSTSGSLSTSTSSSLSGSTSLSTSISKSTSTSASTSSSLSLSTSSS
SSLSGSTSLSTSVSKSTSTSLSNSTASLSASTSTSQSTSTSLSTSASASTSLSTSVSKSTSTSLSNSTASAST
STSISNSLSLSTSLSASTSARTSGSLSLSNSISGSTSSSTSLSTSTSASKSTSSSLSASTSGSRSASTSTSTS
LSSSNSTSLSVSSSLSSSNSLSQSDSQSISSSVSASNSASTSVVNSASASVSQSISGSQSTSNSASLSTSTST
SLSTSASQSASLSQSTSTSRLLSQSVSTSVSESDLTSTSTSLSVSVSQSKETSQSVSTSTSVSESASSSASQS
TSILNSQSLSTSNSQSESQSTSLSLSTSVSSSSSLRQSEENSQSESVSTSTLGSLSDSSSQSESVSNSQSTSD
SESQSSSSSLSLSLSESISGSESLSQSEAVSQSESVSTSTLGSLSDSSSQSESVSSSQSQSDSESQSDSSSLS
DSVSSSISLSTSVFESESTSVSVSTLGSLSDSSSQSESASSSQSKSDSESQSASSSLSLSLSESISSSESLSQ
SEENSQSESISTSTLGSQSDSRSQSESASSSQSTSDSESQSSSSSLSLSLSESISSSESLSQSEAVSQSESVF

TSTLGSLSDSSSQSESVSSSQSTSDSDSQSFSESQSLSFSGSQSGSESFSQSQSESVSLSLSDSKSASASESV
SLSESLSTSVANSTGVADSASVSDSESRSTSISESISKSQSESDAQSDSESISISQSFSHSVTVSESESTSLS
ESLSDSRSLSEFTSTSDSTSISDSLSESLMTSESMSTSEHDSVSTSVSESISVSDSGSLRDSVSLSDSRSLSE
STSSSDSTSISDSLSESVNSSESMSTSEHDSVSTSVSESISVSDSGSLSDSVSQSDSRSVSESTSSSDSTSIS
NSLSESVNSSESMSTSENNSVSTSVSESISVSDSGFLSDSVSQSDSRSLSDSTSVSDSTSTSVSLSNSRKEID
SISASQFILASAIASDSTSVSDSVSTSDFLSQSDSHSLSESTSTSDSTSVSDSLSASLMVSESLSTNIQESVS
TSVSDSISVSDSVSISVSILQSESQSLSDSVYQIESLSMSSSLSDSISSSESLSTSQNQSLSVSVSDSTSVSV
SLLESLSLSQNASQSLSSSGSTSTSLVISDSFSQAISDSMSQSDKDSVLLSISISDSTSVSASMSDSSSLSQD
NSEYVSNSVSGSGSLSESISTSQLTSVSGSDSLSGSHSASDSLSQSLSHSISASDSVSQSLSQSISASLSASS
SSSATTSDSVVVSLSVSGSTSLSTSLSKSTSGSISQSDSQSISTLVSQSASVSVSESLVSSMSISDSLSLSSI
ISNSVSTSQSSSIKTSQSLSNSQSISQSESDSLSTSLSQSAAIVGSETAKTYQIGNFVWEDKNANGIQDAGET
GVAGVTVTLTKPDNTVVSTVSDSNGYYGFDHLIDGQTYTISFETPSGYIPTVADVDPTKDEIDSDGTTVTVTI
NGSDNLTLDSGFVKETYSIGDFVWEDINKNGIQDIGEPGIPGVTVSLTKPDGTTVSKATDVNGFYQFSHLVDG
ETYTISFETPQGYSPTISNVDSRDAIDSDGSTVSVTINGANITSIDSGFVKETYTIGDTVWEDSNKDGIQDAG
EPGIPGVTVTLTNPDGTTVTTTTDANGHYEFTDLPNGDYTVIFETPNGYTPTRSNTGDDTKDSDGQVVKVTVD
GSDNPTIDSGFVKEVHTIGDTVWEDTNKDGIQDAGEPGIPGVTVTLTNPDGTTVTTTTDANGHYEFTDLPNGD
YTVTFETPDGYTPTTSNTGDDTKDSDGQVVKVTVDGSDNPTIDSGFVKVEQPTPGSNSSSESLSQSTTQSSSQ
SSSAKPVASQTAAQLPHTGQAENNGLYGSAALAILAALGLAGKKRNEND*
>group_4009
MSKKQIGSDYVQTDSKSRVKLHKSGKSWVKTTLTSMGLLHLFRGAKTEERVLDTDLDLEAGFSSTHWFKGAAA
LGALVGGAAVTNTAYAAETTSTLPVTSEVATTGTTQVDSTVVASASVSDTTSASASTSASTSQSVSEIQSNSF
SLSTSISESLATSSSISQSTATLNSQALAPKAGQTATDTSKAATTSGLAINEAITSSTITITDTGNYDSNGSL
IPSTIGDGTVLPTSAENIAIDINMTFSDAATAGDQFTIQFSDTVKTDDLAAPGDVYTPIDITDSSGEVIATGT
YNATTKLVTYTFTNYVDKYENVKAVLHLEKYINRDLIQYDTNAVPVTYTVGNNSVTEYFDVKYGLPMTLNNAQ
IESSFSYYDTTNYKVEQIAYVNQDNLNLYNGYIDIFAGANTSGIINTTDTIVEVYRVPAGMQLPDSMEIQDYS
QLEQVPVTIDDRGTNNIGFYLNPNYQYDTSTYVIRVVSSYDPNSTNPIEQFVRLTDGYDTTYVEYSNFIKEAE
DTSGGNGTQSYSIGNYVWVDTNEDGIQNESNTGLAGVLVTLTFPDGSTKGVRTDANGYYEFTGLTDGETYTVS
FETPSGYLPTKVGGGALDDTDSNGLSTTVTINGANDSTLDTGFIIDSTSDSTSTSVSESTSLSDSTSTSTSIS
DSTSESVSTSTSVSDSTSTSDSTSTSESTSTSESTSLSESTSVSDSTSLSESVSTSDSVSVSDSTSVSESTSV
SESTSLSDSVSISDSTSVSDSASISDSTSVSDSANSGSASDSTSTSESTSVSESTSVSDSTSLSESVSTSDSV
SVSDSTSVSESTSVSESTSVSDSTSLSESVSTSDSVSVSDSISVSDSTSVSDSTSLSDSTSVSDSTSASLNSG
SDSDSASVSMSESLSTSTSVSDSTSLSESVSTSDSVLVSDSISVSDSTSVSESTSLSDSVSISDSTSLSESTS
VSDSTSVSDSTSTSDSISVSESSSLSTSISESTSIIGSETSTVYTIGDTVWEDTNKDGIQDAGEPGIPGVTVT
LTNPDGTTVTTTTDANGHYEFTDIPNGDYTVTFETPNGYTPTTSNTGDDTKDSDGQVVKVTVAGSDNPTIDSG
FVKEVHTIGDTVWEDTNKDGIQDAGEPGIPGVTVTLTNPDGTTVTTTTDANGHYEFTDLPNGDYTVTFETPDG
YTPTTSNTGDDTKDSDGQVVKVTVDGSDNPTIDSGFVKEVHTIGDTVWEDTNKDGIQDAGEPGIPGVTVTLTN
PDGTTVTTTTDANGHYEFTDLPNGDYTVTFETPNGYTPTTSNTGDDTKDSDGQVVKVTVDGSDNPTIDSGFVK
VEQSTPGSNSSSESLSQSTTQSSSQSSSAKPVASQTAAQLPHTGQAENNGLYGSAALAILVALGLAGKKRNEN
D*
>group_4104
MNQAKIITGLAVATLSTSVGIVHAEDVTPVTEASTTNETVTVPEVTQSQVDSAKVIADQATSDVNAQQNVVND
AQAQTNQAQSNVVSATTAVKDATATVEQATPEVVTKAQAEVTEATTAVKNAETNLITAQSDVTKVQEAVANQT
QVVAENQTSVDQAQSDVTKAQEAVGSAKQSIDTTKANANLAEAEKLVAEKTTNVATAETSLSEAKQVDAKLAE
EIIQAQATVTQKELAVKDTQDLLNQVASEMSKEQVTTSLQNQSYYNQRDGAWAGYYGNNTFASTGCVPTSLAM
VFTELARRGVTPTEVANYLYNNTNYYNKYFSGTSANGIVSATRAFGFVPTHLDSQNAIAEALQAGHYVVGAVQ
NNKFSPWGPQYSHEVVMRGYSNGNTYVYDPYNRANIGWYPVANLWVERSGDKDDNALGVPFFKITTQKMASIE
TQKAQLTSAVNTAKSQLDQAKQVLSNLQATSLQTPDAQSKLDQAKVELALAQDNYVKAQEAVKLASKELAVKE
ANLKNAQADLLAKQNILKEAQATLAESQLLLASLQDNLKEAQASVADAKTSLDTAKTNLAQKQANLLSLQNAN
KVLTEAQAKLVTAKTDLANKMAILDKEVAKLKELQAVQAEAQNQYSIVFEAFKAVLEAKKQAELTEIYNHIIA
GGGEAIPVVDETGKITGYVDGSQKAVGNEVTLALTSNGQAPLESPVNKENHDLTKTSQVLPHTGEAGVSLLSV
LGAGLISTLGLISLKKRRTH*
>group_4120
MNSNIKGHGYFRKSKAYGLVCGIALAGAFAFNSGNVSADEVTAPATQSQTVVTTLTEPTNNQAQPVTSPALDT
AVDSAKDAGVVVNTTQPVSHTDVPSAQADLANQTQAVKDATAKAEANTQAIKDATAQNAQIDAQNQAEATRVA
EINKAGQLAVDQKNAQAQALVDKQNADAQAQAEATNAQLKADYEAKLAEIKTVEDYNKAVDERNALAKQQAEA
TNAQLKADYQTKLDAYNKALADKINLIANDVSFQGYGKSEDLNALGAVTTDTVTIDGAGNFTLKEPQSDPQGI
IGYITTKGKLNYSSVYDPTTGKAKITIDSITLDTWQLDLTRPSTSGITNASAEYIGLDGTSLFKQAYTGLESI
APITIGKTSAIGQTFTLASGETTPEFMFLKTNPFWQYFAPSSLFAKLTYNTAPLPNKPVEPTLVTVTPEALKP
VPQTPPTPVYVTPTLKSFTPEVYTPIKPTVKPHVAVPDKLVVTVSVHPVMVPVANPSKDVVDQAGKSINGLSV
LPNSDLNYVAQQDFSQYKGMTASNDKVAKNFIYIDDYDEKSLDGKSMVVNSIKASNGQDVSSLLEMHHVLSKD

VLDEKLQAILNNSGLSPVGEFYMWVAKDPSSFFTAYVQKGLDITYNLSFKVNQTFTEGEIVNGVAQIDFGNGY
LGNLVVNDLPKPEVHKDVLDKQDGKSINNGTVKLGDEVTYKLEGWVVPTGRSYDLFEYKFVDQLQNTHDLFLK
DSVVAKVDITLSDGTVITKGSDLAQYTETVYNKETGLYELAFKKSFLTKVVRSSEFGADAFLVVKRVKAGDVY
NDYTLYVNGNLVKSNRVVTHTPEAPKPVTPQKLTPKTPVLPHTGEASMSIVSVIGASIISLLGLSRLRRKVEK
*
